# THBS1^+^ Macrophages Exacerbate Modic Changes via SDC4‐Dependent Activation of NLRP3 Inflammasome

**DOI:** 10.1002/advs.76318

**Published:** 2026-06-26

**Authors:** Xiangxi Kong, Qize Xue, Xiaoan Wei, Jie Li, Bao Huang, Weishao Chen, Zimin Cai, Tao Yang, Chengjun Yao, Jiayan Jin, Bohan Cai, Xuyang Zhang, Junhui Liu, Haihao Wu, Jian Chen, Zhi Shan, Fengdong Zhao

**Affiliations:** ^1^ Department of Orthopaedic Surgery Sir Run Run Shaw Hospital Zhejiang University School of Medicine Hangzhou P. R. China; ^2^ Key Laboratory of Musculoskeletal System Degeneration and Regeneration Translational Research of Zhejiang Province Hangzhou P. R. China; ^3^ Department of Wound Healing The First Affiliated Hospital of Wenzhou Medical University Wenzhou P. R. China; ^4^ Department of Orthopaedic Surgery The Affiliated LiHuiLi Hospital of Ningbo University Ningbo P. R. China; ^5^ School of Medicine Hangzhou City University Hangzhou P. R. China; ^6^ Department of Orthopedics Surgery Ningbo No.2 Hospital Ningbo Zhejiang P. R. China

**Keywords:** cutibacterium acnes, macrophages, modic changes, NLRP3 inflammasome, SDC4, THBS1

## Abstract

Modic changes (MCs) in the lumbar vertebral endplates are a common imaging finding in patients with low back pain. Their pathogenesis and progression are closely linked to the local immune microenvironment, underscoring the need to investigate the immunomodulatory mechanisms involved in MCs. Here, we identified the THBS1^+^ macrophages displaying a strongly pro‐inflammatory phenotype. The THBS1^+^ macrophages were enriched at lesion sites in both human lumbar MCs specimens and the C. acnes‐induced mouse model of MC. In vitro, C. acnes and its key metabolites promoted THBS1 expression in macrophages via Toll‐like receptor (TLR) signaling, while TLR inhibition alleviated MCs by suppressing this macrophage subset. Notably, conditional knockout of THBS1 in macrophages effectively attenuated C. acnes‐induced MCs progression. Mechanistically, we identified syndecan‐4 (SDC4) on endplate chondrocytes as a functional receptor for macrophage‐derived THBS1. THBS1–SDC4 engagement activated p38 phosphorylation, leading to transcriptional upregulation of the NLRP3 inflammasome and amplified inflammatory responses in chondrocytes. Conversely, SDC4 silencing mitigated MCs progression. Collectively, this study delineates a mechanism by which THBS1^+^ macrophages exacerbate lumbar endplate MCs through the SDC4–p38–NLRP3 axis. These findings provide a novel theoretical framework and highlight THBS1 and SDC4 as potential immunotherapeutic targets for modulating disc degeneration.

## Graphical abstract

The illustration of THBS1+ macrophages exacerbate MCs via SDC4‐dependent activation of the NLRP3 inflammasome. THBS1^+^ macrophage subpopulation was identified in MCs through single‐cell RNA sequencing. C. acnes and its metabolites activate THBS1 expression in macrophages via the TLR signaling pathway. The secreted THBS1 protein subsequently activates the NLRP3 inflammasome through the SDC4‐p38 axis, thereby promoting MCs progression. Therapeutic targeting of TLR signaling or silencing of THBS1/SDC4 expression ameliorates MCs.

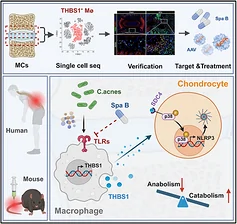

## 1 Introduction

The pathological mechanisms underlying lumbar vertebral endplate degeneration are complex, involving various factors such as mechanical injury [1, 2], low‐virulence infection [3, 4], inflammatory responses [5], and immune dysregulation [6, 7]. Radiographically, endplate degeneration primarily manifests as Modic changes (MCs), most commonly observed in the lower lumbar spine [8]. These changes are characterized by signal intensity abnormalities in the vertebral endplate regions on magnetic resonance imaging (MRI). As a common degenerative pathological alteration, MCs impose a substantial socioeconomic burden, reflected mainly in loss of workforce, consumption of medical resources, and related disability issues, constituting a long‐term public health challenge [9, 10].

Immune homeostasis is essential for maintaining physiological function, and its dysregulation significantly contributes to endplate degeneration [11]. As central immune effectors, macrophages play a critical role in maintaining endplate homeostasis [12]. In response to pathological stimuli like mechanical stress or chronic infection, macrophages in the cartilaginous endplate polarize toward an M1 phenotype, secreting pro‐inflammatory cytokines including IL‐1, IL‐6, and TNF‐α, thereby accelerating degenerative changes [13, 14, 15]. Our previous research found that bony endplates with MCs exhibit regional bone resorption and increased sclerosis, a process potentially regulated by Oncostatin M (OSM) secreted by bone‐resident macrophages, whose activation is influenced by the local immune microenvironment [16]. Additionally, the presence of numerous activated mast cells within Modic lesions further promotes M1 macrophage polarization, hastening disease progression [17]. Collectively, from the acute inflammatory phase to the chronic repair stage, the dynamic interactions within the immune cell network and their crosstalk with the bone microenvironment jointly determine the phenotypic evolution of MCs, the associated clinical pain symptoms, and the disease prognosis.

Thrombospondin‐1 (THBS1) is a multidomain, oligomeric extracellular matrix glycoprotein secreted by various cell types, including macrophages, neutrophils, platelets, and fibroblasts [18]. Its characteristic homotrimeric structure comprises an N‐terminal heparin‐binding domain, a procollagen‐like region, type I repeats, type II epidermal growth factor‐like repeats, and a C‐terminal cell‐binding domain. THBS1 regulates cell adhesion, migration, proliferation, and apoptosis, and contributes to tissue remodeling by activating latent TGF‐β [19], inhibiting angiogenesis [20], and modulating immune‐inflammatory responses [21, 22]. It is implicated in the pathogenesis of various chronic inflammatory and degenerative disorders, such as osteoarthritis [23] and diabetic complications [24, 25, 26, 27], through effects on extracellular matrix metabolism and inflammatory signaling. Nevertheless, the precise role and mechanisms of THBS1 in lumbar endplate Modic changes remain undefined.

This study identifies the enrichment of THBS1^+^ macrophages in MC regions and further elucidates the critical role of this macrophage subset in the pathogenesis and progression of lumbar MCs via the SDC4‐p38‐NLRP3 signaling axis. These findings provide novel insights into the immunoregulatory microenvironment underlying MCs and establish a conceptual foundation for developing immune‐targeted therapeutic strategies against intervertebral disc degeneration by modulating the THBS1/SDC4 pathway.

## 2 Results

### 2.1 Single‐Cell Sequencing of MCs Tissues and Identification of THBS1^+^ Macrophages

To elucidate the role of the immune system in MCs, we established an animal model of MCs by injecting Cutibacterium acnes (C. acnes) into the caudal vertebral endplates of mice [17, 28, 29, 30]. Prior to single‐cell sequencing, the successful establishment of the Modic change rat model was validated by magnetic resonance imaging (MRI) scanning (Figure S1A). Single‐cell RNA sequencing (scRNA‐seq) was performed on the superior and inferior endplate tissues collected 4 weeks post‐modeling (Figure 1A). We identified 15 distinct cell clusters (Figure 1B; Figure S1B,C), including glial cells (marked by *Mpz, Mbp, Pmp22*), granulocyte‐monocyte progenitors (GMPs: *Elane, Prtn3, Mpo*), mesenchymal stem cells (MSCs: *Gsn, Pi15, Cxcl12*), chondrocytes (*Cnmd, Mgp, Col2a1*), endothelial cells (ECs: *Fabp4, Plvap, Igfbp3*), fibroblasts (*Prg4, Saa3, Mmp3*), mural cells (*Rgs5, Acta2, Tagln*), osteoblasts (*Ibsp, Spp1, Mmp13*), tenocytes (*Tnmd, Angptl7, Thbs4*), B cells (*Ighm, Ccr7, Cd83*), T and NK cells (*Ccl5, Xcl1, Ms4a4b*), neutrophils (*S100a9, S100a8, Retnlg*), mononuclear phagocytes (MPs: *Lyz2, Pf4, C1qb*), erythrocytes (*Hbb‐bs, Hba‐a1, Hba‐a2*), and osteoclasts (*Acp5, Mmp9, Ctsk*). The MCs group exhibited significant alterations in immune cell composition, with a sharp increase in the proportion of MPs (Figure 1C).

**FIGURE 1:**
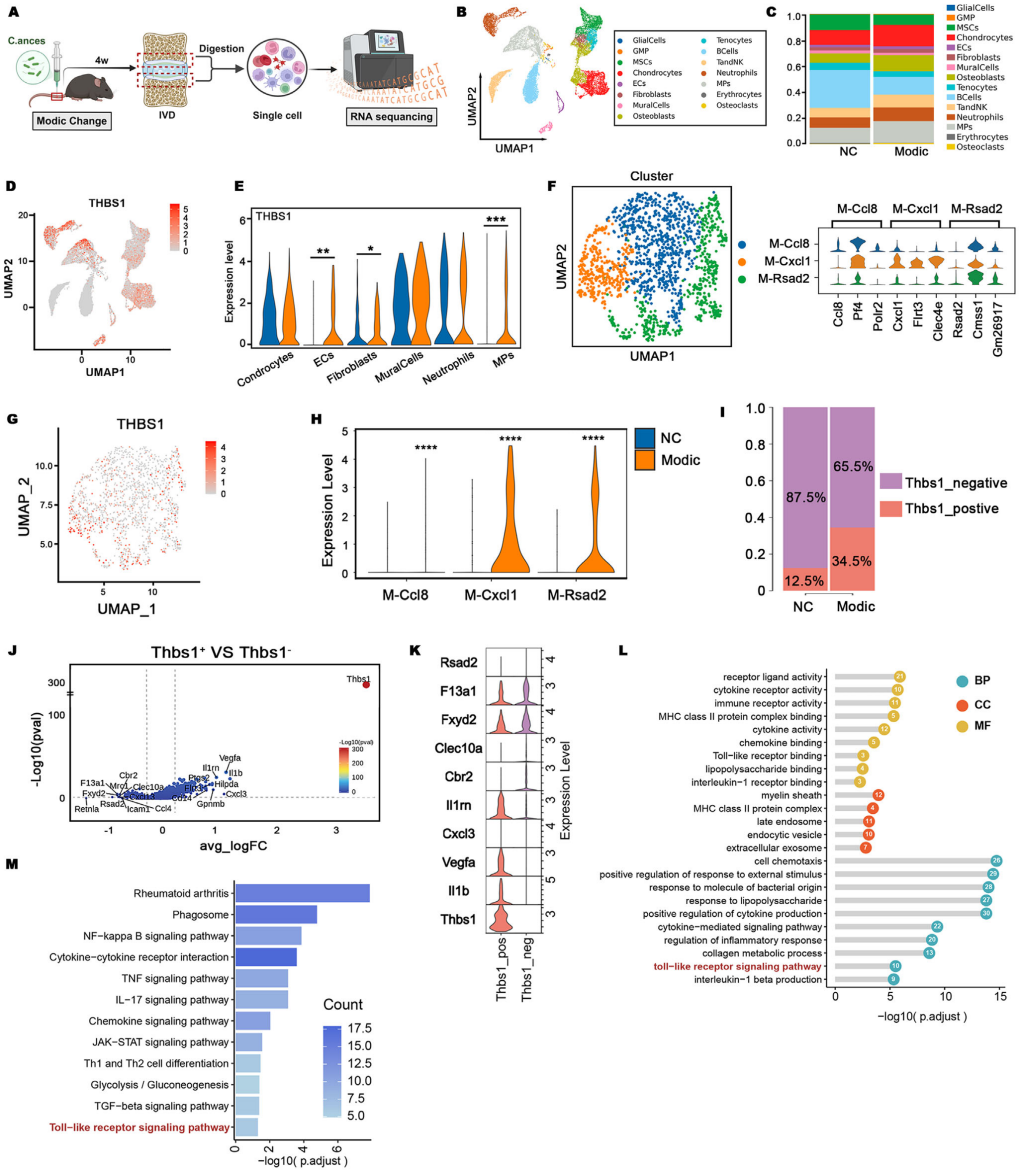
Single‐cell RNA sequencing of MCs tissues identifies THBS1^+^ macrophages. (A) Schematic diagram of single‐cell sequencing of C. acnes‐induced MCs. Following local administration of C. acnes into the caudal vertebral endplate of 8‐week‐old male C57BL/6 mice, the targeted spinal segments were harvested at 4 weeks post‐injection for single‐cell RNA sequencing processing. (B) UMAP plot of single‐cell sequencing of MCs. (C) Proportion of different cell clusters in the NC group and MCs group. (D) UMAP plot showing the distribution of THBS1 across different cell clusters. (E) Violin plots showing the expression differences of THBS1 in Chondrocytes, ECs, Fibroblasts, MuralCells, Neutrophils, and MPs between the NC group and MCs group. (F) UMAP plot and marker genes for the subclustering of MPs clusters. (G) UMAP plot showing the distribution of THBS1 in different MP subpopulations. (H) Violin plots showing the expression differences of THBS1 among different MPs subpopulations. (I) Proportion of THBS1‐positive and THBS1‐negative MPs in the NC group and MCs group. (J) Volcano plot of differentially expressed genes (DEGs) between THBS1^+^ and THBS1^−^ MPs. (K) Marker genes of THBS1^+^ and THBS1^−^ MPs. (L, M) GO and KEGG enrichment analysis of up‐regulated DEGs between THBS1‐positive and THBS1‐negative MPs.

Notably, among the differentially expressed genes in MPs, THBS1 exhibited a pronounced upregulation in the MCs group (Figure S1D). UMAP visualization corroborated that THBS1 was primarily expressed in MPs and neutrophils within the MCs context (Figure 1D). Compared to the control group, THBS1 expression was elevated in MPs of the MCs group, whereas its level in neutrophils showed no statistically significant difference (Figure 1E), suggesting its potential involvement in the pathogenesis of MCs.

Further subclustering of the MPs population revealed three distinct subsets, characterized by specific expression of *Ccl8*, *Cxcl1*, and *Rsad2*, respectively (Figure 1F). The proportion of the *Ccl8* subset decreased in the MCs group, while the *Cxcl1* and *Rsad2* subsets were expanded (Figure S1E). UMAP visualization revealed that THBS1 expression was predominantly localized to the *Cxcl1* and *Rsad2* subsets, where its levels were sharply elevated in MCs (Figure 1G,H). These two subsets exhibited signatures associated with enhanced immune regulation, pro‐angiogenesis, pro‐inflammation, and pro‐proliferation, closely aligning with the pathological alterations in MCs (Figure S1F). We then categorized MPs into THBS1‐positive (THBS1^+^) and THBS1‐negative (THBS1^−^) populations. MP cells with detectable THBS1 expression were classified as the THBS1^+^ subset, while those without or with low THBS1 expression were classified as the THBS1^−^ subset. The THBS1^+^ fraction increased approximately threefold, from 12.5% in the control group to 34.5% in the MCs group (Figure 1I). THBS1^+^ MPs demonstrated a stronger pro‐inflammatory and pro‐angiogenic phenotype, evidenced by higher expression of genes such as *Il1β*, *Il1rn*, *Ptgs2*, *Cxcl3*, and *Vegfa* (Figure 1J,K). Gene Ontology (GO) enrichment analysis of upregulated genes in this population revealed enrichment in biological processes (BP) like cytokine production and immune response; enrichment in Molecular Function (MF) terms related to response to lipopolysaccharide, regulation of inflammatory response, and the Toll‐like receptor signaling pathway. Consistent with this, Kyoto Encyclopedia of Genes and Genomes (KEGG) pathway analysis indicated enrichment in the NF‐κB signaling pathway, TNFα signaling pathway, IL17 signaling pathway, cytokine‐cytokine receptor interaction, and Toll‐like receptor signaling pathway (Figure 1L,M; Figure S1G,H). Collectively, these findings identify THBS1‐positive macrophages with a pro‐inflammatory phenotype as drivers of Modic changes.

### 2.2 C. acnes and Its Metabolites Promote THBS1 Expression in Macrophages

To substantiate the scRNA‐seq findings, we assessed THBS1 expression in human MCs specimens and specimens from a C. acnes‐induced MCs model (Figure 2A). Endplate tissue collected during vertebral fusion surgery was evaluated using MRI (Figure 2B), with surgical specimens from fracture cases serving as controls. Immunofluorescence staining revealed a substantial increase in THBS1‐positive macrophages within the lesion sites (Figure 2C,G). Notably, THBS1^+^ macrophages were also clearly elevated in the C. acnes‐induced MCs model compared to the sham group (Figure 2D–F,H). These consistent results across species establish THBS1^+^ macrophages as a pivotal contributor to MCs pathogenesis.

**FIGURE 2:**
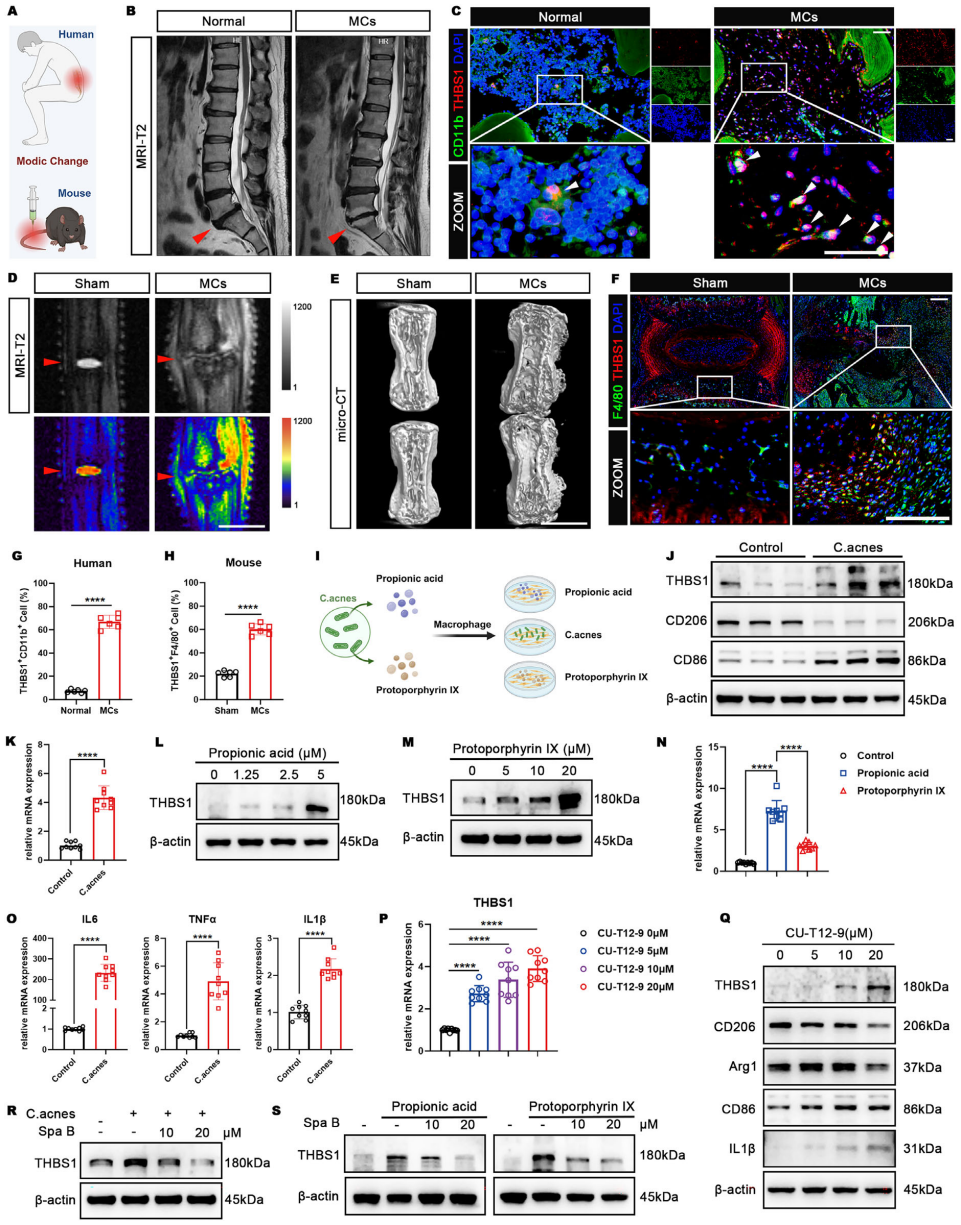
C. acnes and Its Metabolites Promote THBS1 Expression in Macrophages. (A) Clinical MCs specimens and the C. acnes‐induced MCs model. (B) MRI images of intervertebral disc herniation and endplate Modic changes in a typical clinical patient. The affected segments are marked by red arrows. (C) Representative THBS1 immunofluorescence staining images of endplate tissues from the Normal and MCs groups. Green: CD11b (a pan‐macrophage/myeloid marker); Red: THBS1; Blue: DAPI. White arrows indicate THBS1^+^CD11b^+^ double‐positive cells. Scale bar = 40 µm. (D) Representative images of magnetic resonance imaging (MRI) of C. acnes‐induced modic changes. The corresponding pseudocolor images were shown below. Scale bar = 1 mm. The affected segments are marked by red arrows. (E) Representative images of micro‐CT of C. acnes‐induced modic changes. Scale bar = 1 mm. (F) Representative THBS1 immunofluorescence staining images from the Sham group and C. acnes‐induced MCs group. Green: F4/80; Red: THBS1; Blue: DAPI. Scale bar = 160 µm. (G) Proportion of THBS1^+^CD11b^+^ double‐positive cells (*n* = 6). (H) Proportion of THBS1^+^F4/80^+^ double‐positive cells (*n* = 6). (I) Schematic diagram of the co‐culture system involving C. acnes or its metabolites (Propionic Acid and Protoporphyrin IX) with macrophages. (J) Western blot of THBS1 protein levels and polarization markers in macrophages stimulated with C. acnes for 24 h. CD206 is a marker for M2‐type macrophages, while CD86 is a marker for M1‐type macrophages. (K) RT‐qPCR of THBS1 gene expression in macrophages stimulated with C. acnes for 24 h (*n* = 9). (L) Western blot of THBS1 protein levels in macrophages treated with a concentration gradient of Propionic Acid for 24 h. (M) Western blot of THBS1 protein levels in macrophages treated with a concentration gradient of Protoporphyrin IX for 24 h. (N) RT‐qPCR of THBS1 gene expression in macrophages treated with 5 µM Propionic acid or 20 µM Protoporphyrin IX for 24 h (*n* = 9). (O) RT‐qPCR of TLRs target genes in BMMs stimulated with C. acnes for 24 h (*n* = 9). (P) RT‐qPCR of THBS1 gene levels in BMMs treated with different concentrations of TLR2 agonist CU‐T12‐9 for 24 h (*n* = 9). (Q) WB of THBS1 and M1 polarization markers in BMMs stimulated with different concentrations of TLR2 agonist CU‐T12‐9 for 24 h. (R) Western blot of THBS1 protein levels in C. acnes‐stimulated macrophages treated with the TLRs inhibitor Sparstolonin B for 24 h. 10µm as the low‐dose group and 20µm as the high‐dose group. (S) Western blot of THBS1 protein levels in macrophages stimulated with propionic acid or protoporphyrin IX and treated with the TLR2/4 inhibitor Sparstolonin B for 24 h. Statistical analyses were determined by two‐tailed Student's *t*‐test (G, H, K, O) and one‐way ANOVA (N, P). ns indicated no statistical difference, ^*^ *p* < 0.05, ^**^ *p* < 0.01, ^***^ *p* < 0.001, and ^****^ *p* < 0.0001. Data were presented as mean ± SD.

Having established this association in vivo, we asked whether C. acnes infection directly regulates THBS1 in macrophages. To this end, we stimulated bone marrow‐derived macrophages (BMMs) with C. acnes or its key metabolites, propionate and protoporphyrin IX (Figure 2I). The bacteria potently induced both THBS1 expression and M1‐like polarization (Figure 2J,K). Additionally, the metabolites alone were sufficient to elicit a concentration‐dependent upregulation of THBS1 (Figure 2L–N), identifying them as active mediators in this process.

### 2.3 TLR2/4 Antagonism Suppresses Macrophage THBS1 Expression and Alleviates MCs

Previous studies have demonstrated that C. acnes itself and its cell wall components (e.g., lipoteichoic acid and peptidoglycan) can activate TLR2 [31]. In addition, other reports indicate that C. acnes is capable of activating macrophage M1 polarization and promoting the secretion of inflammatory cytokines [32, 33, 34]. As shown in our Figure 1L,M, TLR signaling pathways are activated in THBS1^+^ macrophages, suggesting a significant correlation between C. acnes and TLRs. Based on these lines of evidence, our hypothesis posited that C. acnes activates macrophages, which are recognized by Toll‐like receptors (TLR2 and TLR4), leading to THBS1 upregulation. To test this hypothesis, we first examined the expression levels of TLR target genes (*Il6, Tnfα, Il1β*) and macrophage polarization markers in C. acnes‐stimulated macrophages. Following C. acnes stimulation, the levels of *Il6, Tnfα, Il1β*, and M1 marker genes (*iNOS, CXCL1, CD80, CD86*) were significantly upregulated, whereas M2 markers (*CD206, ARG1, CD163*) were significantly downregulated, suggesting that C. acnes activates the TLR signaling pathway (Figure 2O; Figure S2A). To determine whether C. acnes regulates THBS1 expression through the TLR signaling pathway, we applied the TLR2 agonist CU‐T12‐9. The results showed that CU‐T12‐9 significantly upregulated THBS1 gene and protein levels (Figure 2P,Q; Figure S2B). Furthermore, we employed Sparstolonin B, a dual TLR2/4 inhibitor. Treatment with Sparstolonin B effectively suppressed THBS1 upregulation in C. acnes‐stimulated macrophages (Figure 2R; Figure S2C), confirming the TLR dependence of this process. Furthermore, Sparstolonin B also reversed the increase in THBS1 expression induced by propionate and protoporphyrin IX in macrophages (Figure 2S).

We next investigated whether TLRs inhibition could mitigate MCs progression in vivo. WT mice were subjected to MCs induction and treated with Sparstolonin B (Figure 3A). MRI and micro‐CT analyses revealed that C. acnes induction led to characteristic MCs features, including diminished disc signal intensity, bony endplate erosion, and aberrant ossification. These degenerative changes were significantly ameliorated by Sparstolonin B in a dose‐dependent manner (Figure 3B,C). Histological assessment (H&E, Safranin O/Fast Green, and Masson staining) confirmed that Sparstolonin B reduced cartilage endplate destruction and inflammatory cell infiltration (Figure 3D,E; Figure S3A). Consistent with our in vitro findings, Sparstolonin B also suppressed THBS1 expression in macrophages in vivo (Figure 3F,G).

**FIGURE 3:**
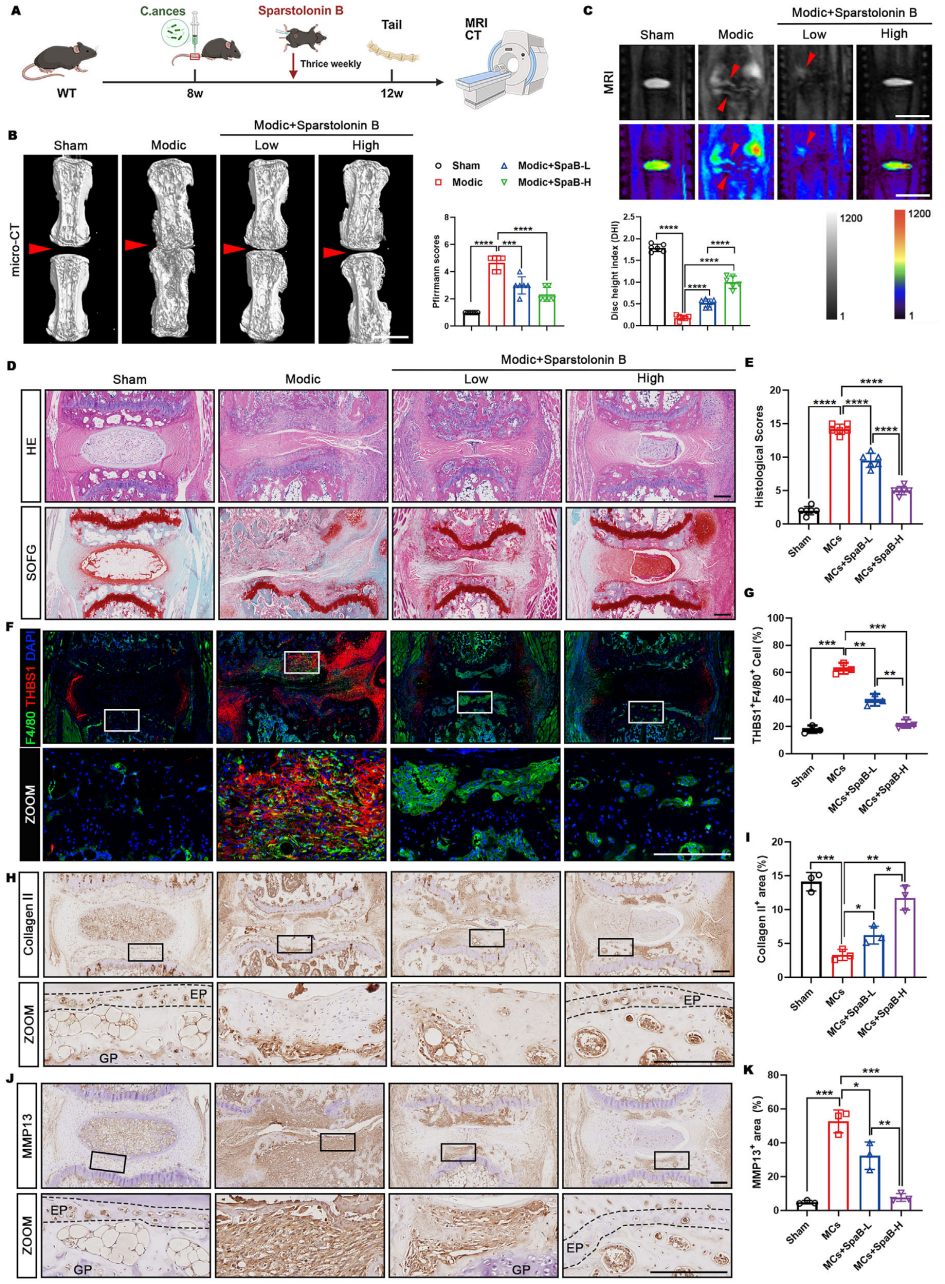
TLR2/4 Antagonism Suppresses Macrophage THBS1 Expression and Alleviates MCs. (A) Schematic illustration of in vivo Sparstolonin B treatment ameliorating C. acnes‐induced MCs. 8‐week‐old male C57BL/6 mice were injected with C. acnes in the tail vertebra and administered low‐ and high‐dose Sparstolonin B via intraperitoneal injection three times per week for 4 weeks. (B) Representative micro‐CT images along with the disc height index and Pfirrmann grade (*n* = 6). The affected segments are marked by red arrows. Scale bar = 1 mm. (C) Representative images of magnetic resonance imaging (MRI). The corresponding pseudocolor images were shown below. Typical features of Modic changes are marked with red arrows. Scale bar = 1 mm. (D, E) Representative images of hematoxylin and eosin (H&E) and safranin O/fast green (SOFG) staining, along with the histological scores (*n* = 6). Scale bar = 200 µm. (F, G) Representative immunofluorescence staining images for THBS1 and corresponding statistical analysis (*n* = 3). Scale bar = 200 µm. (H, I) Representative immunohistochemical staining images for Collagen II and statistical analysis of the percentage of positive area (*n* = 3). Scale bar = 200 µm. (J, K) Representative immunohistochemical staining images for MMP13 and statistical analysis of the percentage of positive area (*n* = 3). Scale bar = 200 µm. Statistical analyses were determined by one‐way ANOVA (B, E, G, I, K). ns indicated no statistical difference, ^*^ *p* < 0.05, ^**^ *p* < 0.01, ^***^ *p* < 0.001, and ^****^ *p* < 0.0001. Data were presented as mean ± SD.

The C. acnes‐induced MCs group exhibited a matrix catabolic shift, characterized by downregulated collagen II (an anabolic marker) and upregulated MMP13 (a catabolic marker), which was reversed by Sparstolonin B treatment (Figure 3H–K). Furthermore, Sparstolonin B potently inhibited the pathological ingrowth of blood vessels and nerves into the endplate (Figure S3B,C), an effect likely attributable to reduced inflammatory cytokine and chemokine release following TLRs blockade. Consequently, these results demonstrate that TLRs antagonism alleviates the progression of MCs by inhibiting the C. acnes‐induced upregulation of THBS1 in macrophages.

### 2.4 Macrophage‐Specific THBS1 Knockout Alleviates C. acnes‐Induced MCs

To definitively elucidate the role of macrophage‐derived THBS1 in MCs, we generated macrophage‐specific THBS1 knockout (THBS1 KO) mice by crossing THBS1*^flox/flox^* mice with Lysm‐*Cre* mice. Littermates carrying the THBS1*^flox/flox^* allele without Cre were used as controls. No gross morphological differences were observed between the THBS1 KO and WT mice (Figure S4A,B). Immunofluorescence staining confirmed the nearly complete absence of THBS1 signal in macrophages of the knockout mice (Figure S4C,D). To investigate the effect of THBS1 knockout on resting macrophages, we extracted bone marrow from WT and THBS1 KO mice for flow cytometry analysis. The results indicated that THBS1 knockout reduced the proportion of macrophages in mouse bone marrow, slightly increased the proportion of M1‐type macrophages, but had no effect on the proportion of M2‐type macrophages (Figure S4F–K). At the gene level, THBS1 knockout exhibited varying degrees of influence on macrophage polarization markers (Figure S4L).

To further investigate the role of THBS1 in macrophages in the context of Modic changes, we performed transcriptome sequencing of THBS1 knockout mouse‐derived BMMs following stimulation with C. acnes. Principal component analysis (PCA), volcano plots, and heatmaps revealed marked differences in the global gene expression profiles of macrophages after THBS1 knockout (Figure S5A–C). A chord plot of the downregulated differentially expressed genes (DEGs) indicated that genes involved in regulating inflammatory responses were significantly downregulated (Figure S5D). GO enrichment analysis showed enrichment of terms such as cellular response to lipopolysaccharide, inflammatory response, CXCR chemokine receptor binding, and cytokine activity (Figure S5E). KEGG pathway enrichment analysis further revealed enrichment of the TNF‐α signaling pathway, cytokine–cytokine receptor interaction, and the IL17 signaling pathway (Figure S5F). Moreover, GSEA suggested that THBS1 knockout macrophages exhibited reduced metabolism of propanoate, a metabolite of C. acnes (Figure S5G). Collectively, these transcriptomic results suggest that THBS1 regulates the inflammatory response of macrophages and contributes to the progression of Modic changes.

We then induced MCs in 8‐week‐old THBS1 KO and WT mice via C. acnes injection, performing MRI scans and collecting coccygeal vertebral samples 4 weeks post‐modeling (Figure 4A). MRI revealed that THBS1 deletion strikingly ameliorated the C. acnes‐induced reduction in disc signal intensity and inflammatory edema (Figure 4B). Micro‐CT 3D reconstruction demonstrated that THBS1 knockout mitigated bony endplate destruction, ectopic ossification, and disc height loss, as reflected by significantly lower Pfirrmann grades and higher disc height index compared to controls (Figure 4C–E).

**FIGURE 4:**
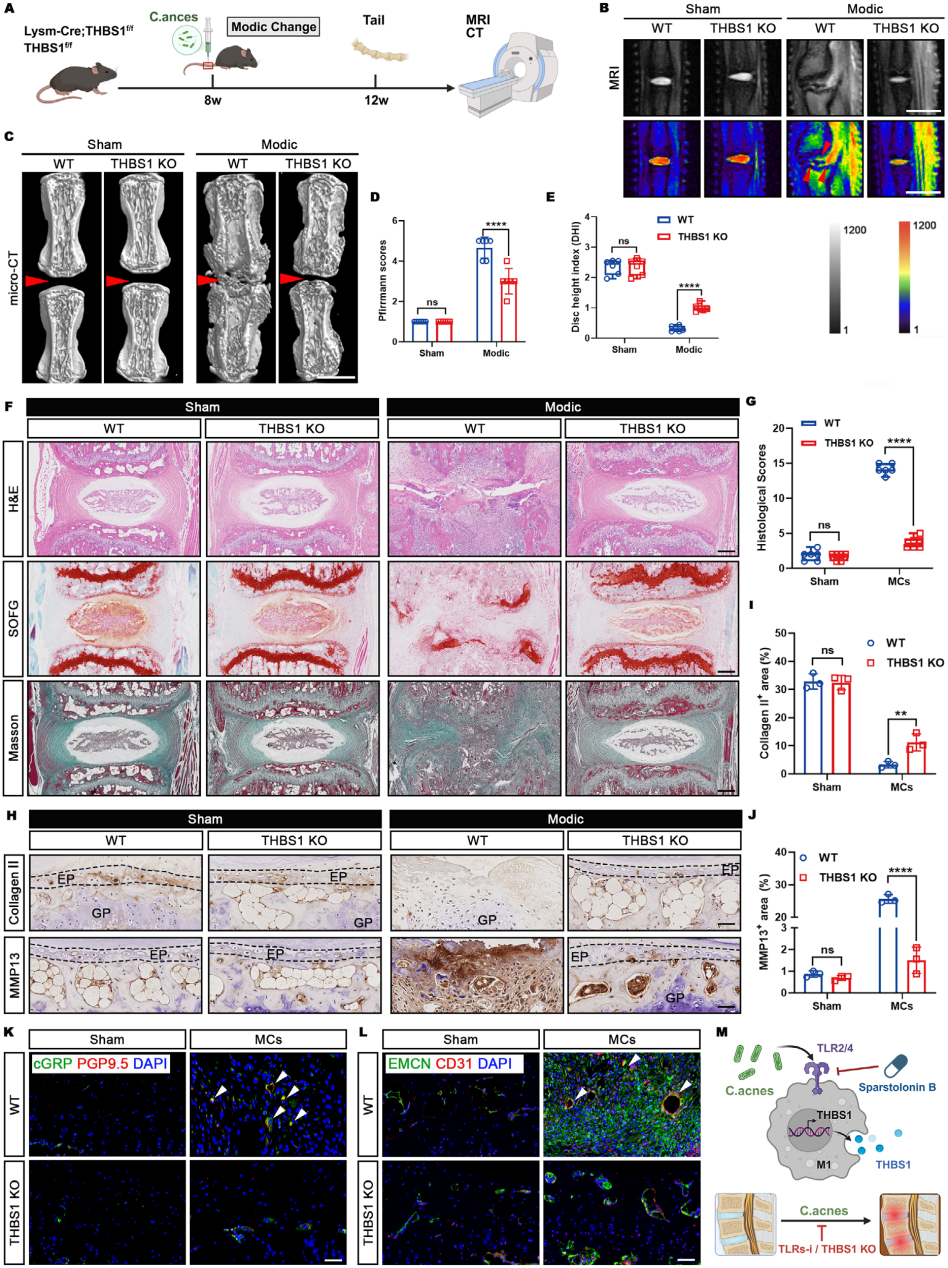
Macrophage‐specific THBS1 knockout alleviates C. acnes‐induced MCs. (A) Schematic illustration of in vivo Macrophage‐specific THBS1 knockout ameliorating C. acnes‐induced MCs. 8‐week‐old male THBS1 *^flox/flox^* (WT) and Lysm‐*Cre*; THBS1 *^flox/flox^* (THBS1 KO) mice were injected with C. acnes in the tail vertebra. (B) Representative images of MRI. The corresponding pseudocolor images were shown below. Typical features of Modic changes are marked with red arrows. Scale bar = 1 mm. (C–E) Representative micro‐CT images along with the disc height index and Pfirrmann grade (*n* = 6). The affected segments are marked by red arrows. Scale bar = 1 mm. (F, G) Representative images of hematoxylin and eosin (H&E), safranin O/fast green (SOFG), and Masson staining, along with the histological scores (*n* = 6). Scale bar = 200 µm. (H‐J) Representative immunohistochemical staining images for Collagen II and MMP13 and statistical analysis of the percentage of positive area (*n* = 3). Scale bar = 40 µm. (K, L) Representative images and statistical analysis of tissue immunofluorescence staining for vascular endothelial cells (CD31 and EMCN) and nerve fiber (cGRP and PGP9.5). White arrows indicate double‐positive cells. Scale bar = 40 µm. (M) Schematic Diagram: C. acnes exacerbates the progression of MC alterations by regulating THBS1 transcription and translation via TLRs, which can be alleviated by targeting TLRs with Sparstolonin B or through macrophage‐specific THBS1 knockout. Statistical analyses were determined by two‐way ANOVA (D, E, G, I, J). ns indicated no statistical difference, ^*^ *p* < 0.05, ^**^ *p* < 0.01, ^***^ *p* < 0.001, and ^****^ *p* < 0.0001. Data were presented as mean ± SD.

Consistent with imaging observations, THBS1 knockout markedly attenuated cartilaginous endplate degradation and inflammatory cell infiltration, leading to improved histological scores (Figure 4F,G). Furthermore, THBS1 deficiency promoted Collagen II and suppressed MMP13 (Figure 4H–J; Figure S6A). We also observed a significant reduction in local neovascularization (CD31^+^EMCN^+^) and nerve fiber ingrowth (cGRP^+^PGP9.5^+^) within the discs of knockout mice (Figure 4K,L; Figure S6B), indicating that THBS1 ablation likely alleviates pathological pain. Collectively, our in vivo studies indicate that THBS1 production by macrophages critically promotes MC progression. Thus, interventions targeting either upstream TLRs or THBS1 itself can mitigate disease severity, highlighting the therapeutic potential of this pathway in attenuating disc degeneration (Figure 4M).

### 2.5 Identification of SDC4 on Chondrocytes as the Key Receptor of THBS1

Given its role as a multifunctional secreted protein, we next sought to identify the target cells and cognate receptors for THBS1. CellChat analysis of intercellular communication revealed extensive interactions involving myeloid cells (Figure S7A), underscoring a pivotal role for macrophages in MCs. Ligand‐receptor interaction analysis identified the THBS1‐SDC4 pair as the most significantly altered in MCs, with interactions predominantly occurring between macrophages and chondrocytes (Figure 5A). Further analysis of the THBS signaling pathway confirmed robust communication between these cell types (Figure 5B). UMAP visualization and feature plotting showed that SDC4 was primarily expressed within the chondrocyte cluster (Figure 5C), which was further supported by dot plot analysis across cell types (Figure 5D). Thus, we identify SDC4 on chondrocytes as the principal receptor mediating THBS1 signaling.

**FIGURE 5:**
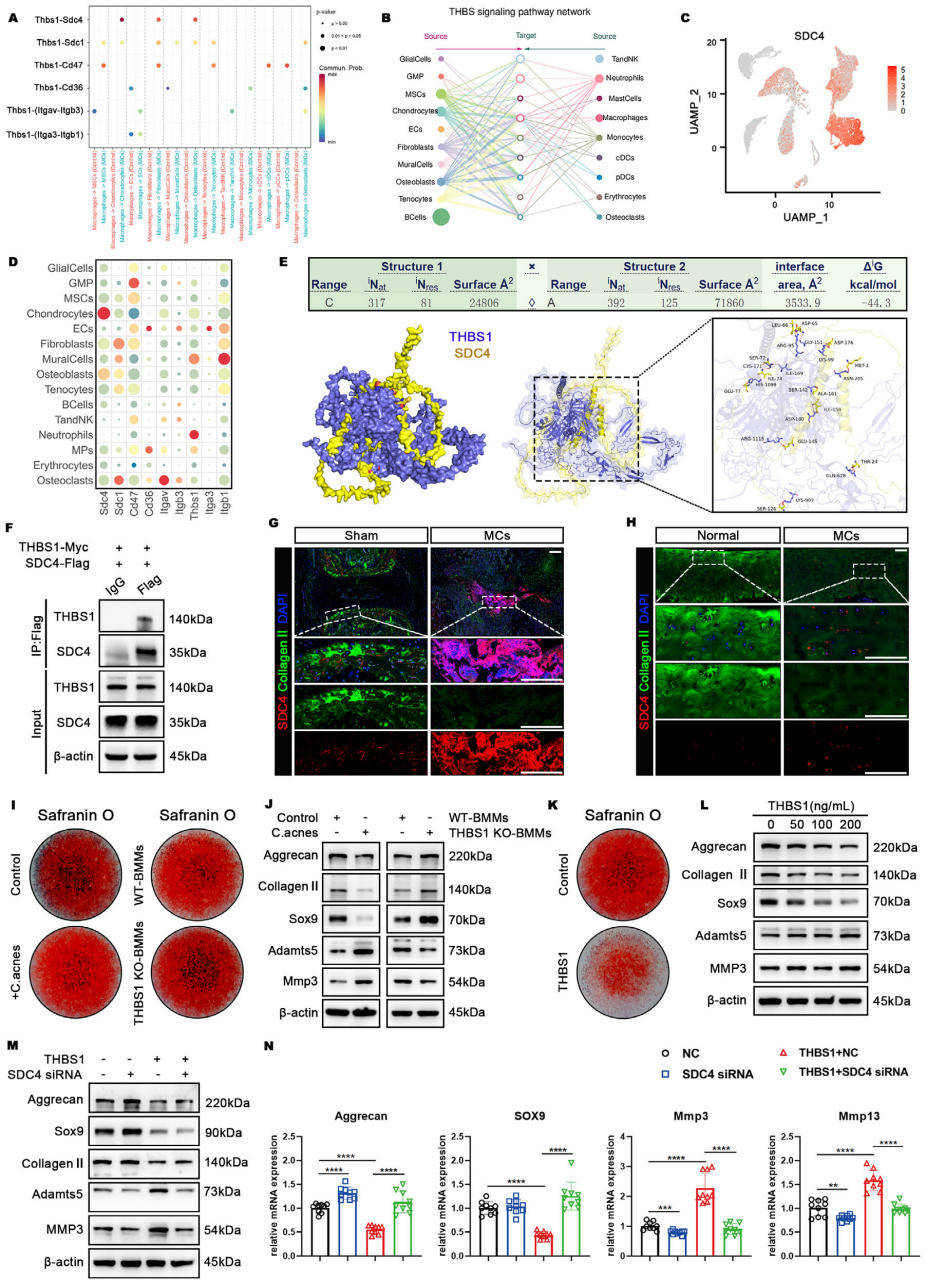
Identification of SDC4 on Chondrocytes as the Key Receptor of THBS1. (A) Receptor‐ligand pair analysis of the Control and MCs groups. (B) THBS1 signaling pathway network among different cell clusters. (C) UMAP plot showing the distribution of SDC4 across cell clusters. (D) Dot plot illustrating the expression abundance of THBS1 and its receptors in different cell clusters. (E) Molecular docking simulation between THBS1 and SDC4. (F) Co‐immunoprecipitation (Co‐IP) of THBS1 and SDC4. Hela cells were transfected with plasmids encoding THBS1‐Myc and SDC4‐Flag, and proteins were harvested for Co‐IP 48 h post‐transfection. (G) Representative images of fluorescence intensity for SDC4 and Collagen II immunofluorescence staining in mouse MCs. Scale bar = 40 µm. (H) Representative images and statistical analysis of fluorescence intensity for SDC4 and Collagen II immunofluorescence staining in clinical endplate cartilage specimens. Scale bar = 40 µm. (I) Safranin O staining of endplate chondrocytes in high‐density culture. Endplate chondrocytes were seeded at high density in the lower chamber of 6‐well plates, with C. acnes‐stimulated macrophages or THBS1‐knockout macrophages seeded in the upper transwell inserts. After 48 h of co‐culture, the lower chondrocytes were subjected to Safranin O staining. (J) Western blot of anabolic and catabolic markers in chondrocytes co‐cultured with macrophages. (K) Safranin O staining of endplate chondrocytes in high‐density culture treated with recombinant THBS1 protein. (L) Western blot analysis of anabolic and catabolic markers in chondrocytes treated with recombinant THBS1 protein. (M) Western blot of anabolic and catabolic markers in chondrocytes transfected with SDC4 siRNA and treated with recombinant THBS1 protein. (N) RT‐qPCR of anabolic and catabolic markers in chondrocytes transfected with SDC4 siRNA and treated with recombinant THBS1 protein (*n* = 9). Statistical analyses were determined by two‐tailed Student's *t*‐test (G, H) and one‐way ANOVA (N). ns indicated no statistical difference, ^*^ *p* < 0.05, ^**^ *p* < 0.01, ^***^ *p* < 0.001, and ^****^ *p* < 0.0001. Data were presented as mean ± SD.

To validate this interaction, we transfected THBS1‐Myc and SDC4‐Flag plasmids into HeLa cell lines, and co‐immunoprecipitation (Co‐IP) assays confirmed a direct binding between THBS1 and SDC4 proteins (Figure 5F). Molecular docking predicted a strong binding affinity, with a binding free energy of −44.3 kcal/mol, indicating the formation of a stable complex. The interaction was stabilized by hydrogen bonds at multiple residues, with key amino acids involved in the binding interface illustrated in Figure 5E. Notably, the expression of SDC4 was upregulated in both human MCs specimens and the C. acnes‐induced mouse model, while the fluorescence intensity of collagen II was reduced (Figure 5G,H).

We next employed a macrophage–endplate chondrocyte co‐culture system to study the regulatory effect of THBS1 derived from macrophages (Figure S7B,D). Under high‐density culture conditions, macrophages stimulated with C. acnes were found to aggravate the degeneration of endplate chondrocytes (Figure 5I). Conversely, compared to WT macrophages, THBS1‐knockout macrophages attenuated proteoglycan loss in the chondrocytes. Corroborating the Safranin O staining, Western blot and RT‐qPCR analyses showed that C. acnes‐stimulated macrophages inhibited matrix anabolism and enhanced catabolism, while THBS1‐deficient macrophages exhibited chondroprotective properties (Figure 5J; Figure S7C,E).

Furthermore, recombinant THBS1 protein accelerated proteoglycan loss in endplate chondrocytes (Figure 5K). Specifically, treatment with recombinant THBS1 induced a concentration‐dependent shift toward a matrix catabolic phenotype, while knockdown of SDC4 rescued this effect (Figure 5L–N; Figure S7F). Thus, we have identified and validated SDC4 on chondrocytes as the functional receptor for macrophage‐derived THBS1, and their interaction exacerbates the progression of MCs.

### 2.6 The SDC4‐p38‐NLRP3 Inflammasome Axis Promotes Degeneration of Endplate Chondrocytes

Syndecan‐4 (SDC4) is a transmembrane heparan sulfate proteoglycan comprised of an extracellular domain capable of binding glycosaminoglycan chains, a transmembrane domain, and a cytoplasmic domain [35]. It functions as a co‐receptor that works in concert with adhesion receptors such as integrins to regulate cell‐matrix adhesion [36], focal adhesion formation [37, 38], and cytoskeletal organization [39]. To investigate the intracellular signaling events following THBS1 binding and activation of SDC4, we treated endplate chondrocytes with recombinant THBS1 (rTHBS1), with or without SDC4 siRNA knockdown, and performed RNA sequencing (RNA‐seq) to profile global gene expression changes (Figure 6A; Figure S8A–C).

**FIGURE 6:**
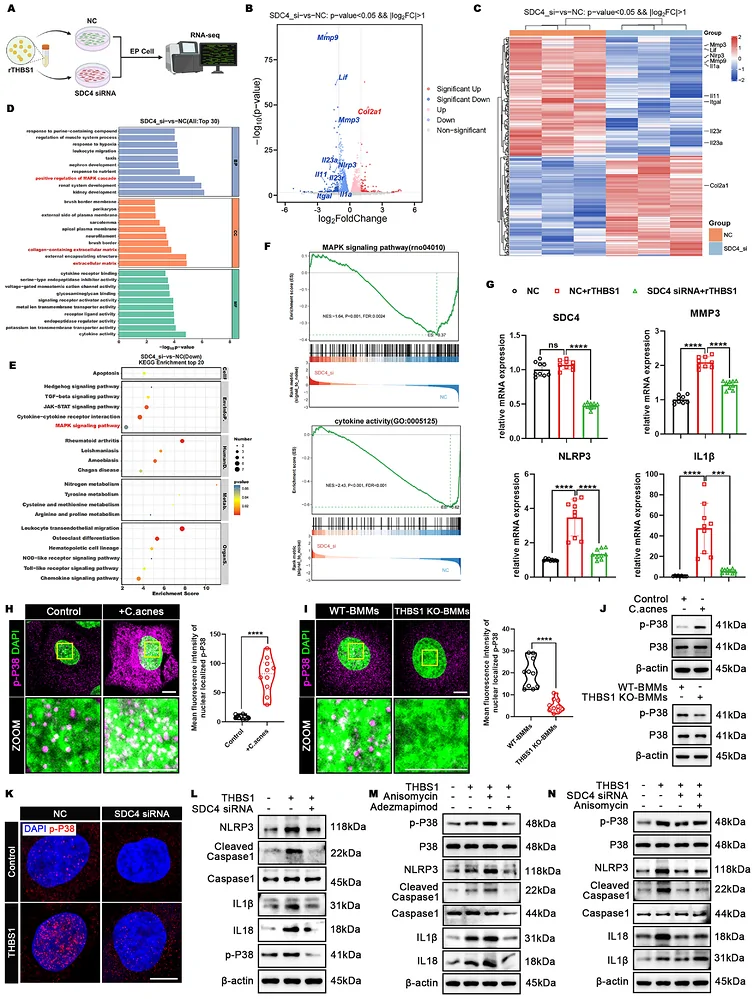
The SDC4‐p38‐NLRP3 Inflammasome Axis Promotes Degeneration of Endplate Chondrocytes. (A) Schematic of the experimental design in which SDC4 siRNA‐transfected endplate chondrocytes were treated with recombinant THBS1 protein for RNA sequencing. (B) Volcano plot of differentially expressed genes. (C) Heatmap of differentially expressed genes. (D) GO enrichment analysis of all differentially expressed genes. (E) KEGG enrichment analysis of down‐regulated differentially expressed genes. (F) GSEA enrichment analysis for MAPK signaling pathway and cytokine activity. (G) RT‐qPCR of inflammatory cytokine gene levels in endplate chondrocytes treated with recombinant THBS1 protein following SDC4 siRNA transfection (*n* = 9). (H) Representative immunofluorescence images of nuclear p‐p38 staining and quantitative analysis of nuclear p‐p38 fluorescence intensity in endplate chondrocytes co‐cultured with C. acnes‐stimulated macrophages (*n* = 10). Scale bar = 5 µm. (I) Representative immunofluorescence images of nuclear p‐p38 staining and quantitative analysis of nuclear p‐p38 fluorescence intensity in endplate chondrocytes co‐cultured with THBS1‐KO macrophages (*n* = 10). Scale bar = 5 µm. (J) Western blot of the p38 signaling pathway in endplate chondrocytes co‐cultured with either C. acnes‐stimulated or THBS1‐KO macrophages. (K) Representative immunofluorescence images of nuclear p‐p38 staining in SDC4 siRNA‐transfected endplate chondrocytes treated with recombinant THBS1 protein. Scale bar = 5 µm. (L) Western blot of the NLRP3 inflammasome in SDC4 siRNA‐transfected endplate chondrocytes treated with recombinant THBS1 protein. (M) Western blot of the NLRP3 inflammasome in endplate chondrocytes treated with recombinant THBS1 protein and supplemented with a p38 agonist (anisomycin, 100nM) or a p38 inhibitor (adezmapimod, 20nM). (N) Western blot analysis of the NLRP3 inflammasome in SDC4 siRNA‐transfected endplate chondrocytes treated with recombinant THBS1 protein and the p38 agonist anisomycin. Statistical analyses were determined by two‐tailed Student's *t*‐test (H, I) and one‐way ANOVA (G). ns indicated no statistical difference, ^*^ *p* < 0.05, ^**^ *p* < 0.01, ^***^ *p* < 0.001, and ^****^ *p* < 0.0001. Data were presented as mean ± SD.

Consistent with our previous findings, SDC4 knockdown led to the upregulation of *Col2a1* and downregulation of matrix metalloproteinases (*Mmp3, Mmp9*), as well as several inflammatory cytokines and receptors (*Lif, Il1a, Il11, Il23a, Il23r*). Notably, we observed a significant downregulation of the NLRP3 inflammasome upon SDC4 silencing (Figure 6B,C). GO and KEGG analyses of the differentially expressed genes further revealed significant enrichment in the MAPK signaling pathway and extracellular matrix organization (Figure 6D,E; Figure S8D–F). Supporting this, Gene Set Enrichment Analysis (GSEA) indicated a coordinated downregulation of the MAPK signaling pathway and cytokine activity (Figure 6F; Figure S8G,H). Previous studies have suggested that the MAPK signaling pathway is closely associated with the NLRP3 inflammasome [40, 41]. We hypothesized that THBS1 is recognized by the SDC4 receptor and subsequently activates the NLRP3 inflammasome via activation of the MAPK signaling pathway. Consistent with this hypothesis, we found that SDC4 siRNA attenuated the THBS1‐induced upregulation of *Mmp3, Nlrp3, and Il1β* (Figure 6G).

Next, we investigated the MAPK signaling pathway and observed that THBS1 treatment increased the phosphorylation level of p38, whereas silencing of SDC4 reversed this effect (Figure S9A). These findings suggest that p38 may act as a downstream component of the THBS1–SDC4 axis. Accordingly, in co‐culture systems, C. acnes‐activated macrophages enhanced p38 phosphorylation and nuclear localization in endplate chondrocytes, while THBS1‐KO macrophages inhibited these changes (Figure 6H–J; Figure S9B,C).

Based on this, we hypothesized that the THBS1‐SDC4 axis regulates NLRP3 transcription via p38. THBS1 treatment enhanced both p38 phosphorylation and its nuclear localization, whereas SDC4 knockdown reduced these effects (Figure 6K; Figure S9A). Western blot further confirmed that THBS1 promoted p38 phosphorylation and upregulated NLRP3 protein levels (Figure 6L). The NLRP3 inflammasome, upon oligomerization, recruits the adaptor protein ASC, leading to the activation of caspase‐1. Active caspase‐1 then proteolytically cleaves the inactive precursors of IL‐1β and IL‐18 into their mature, biologically active forms [42]. We found that rTHBS1 promoted caspase‐1 activation and elevated the levels of IL‐1β and IL‐18, which were rescued by SDC4 siRNA. We next employed the p38 agonist anisomycin and the inhibitor adezmapimod to determine whether p38 is the critical pathway through which THBS1‐SDC4 regulates NLRP3 expression and activation. Anisomycin treatment upregulated and activated NLRP3, thereby promoting the maturation of IL‐1β and IL‐18, whereas adezmapimod produced the opposite effect (Figure 6M). Furthermore, anisomycin was able to reverse the suppressive effects of SDC4 siRNA (Figure 6N). Safranin O staining of high‐density cultured endplate chondrocytes indicated that the p38 agonist exacerbated THBS1‐induced proteoglycan loss, whereas the p38 inhibitor attenuated this effect (Figure S9D). SDC4 knockdown rescued THBS1‐induced proteoglycan loss, and this rescue effect was inhibited by the p38 agonist (Figure S9E). Collectively, these results demonstrate that the THBS1‐SDC4‐p38‐NLRP3 axis regulates the inflammatory response and promotes the degeneration of endplate chondrocytes.

### 2.7 Targeting SDC4 Alleviates MCs Progression

Based on the identification of SDC4 as a key receptor for THBS1, we engineered an AAV vector expressing shRNA targeting SDC4 under the control of the Col2a1 promoter to achieve chondrocyte‐specific knockdown. 8‐week‐old WT mice received an intradiscal injection of this AAV‐shRNA vector via a micro‐syringe (0.26 mm needle). Two weeks later, MCs were induced at the same disc level by injection of C. acnes. Vertebral samples were collected for analysis 4 weeks post‐induction (Figure 7A). Immunofluorescence staining confirmed a substantial reduction in SDC4 fluorescence intensity in the AAV‐shSDC4 group compared to controls (Figure S10A,B). MRI revealed a complete loss of disc signal intensity in the MCs group, whereas the AAV‐shSDC4 group exhibited a notable rescue effect, with better‐preserved disc integrity and reduced endplate edema (Figure 7B). Micro‐CT further demonstrated that SDC4 knockdown mitigated bony endplate destruction, reduced disc height loss, and improved Pfirrmann grades (Figure 7C,D).

**FIGURE 7:**
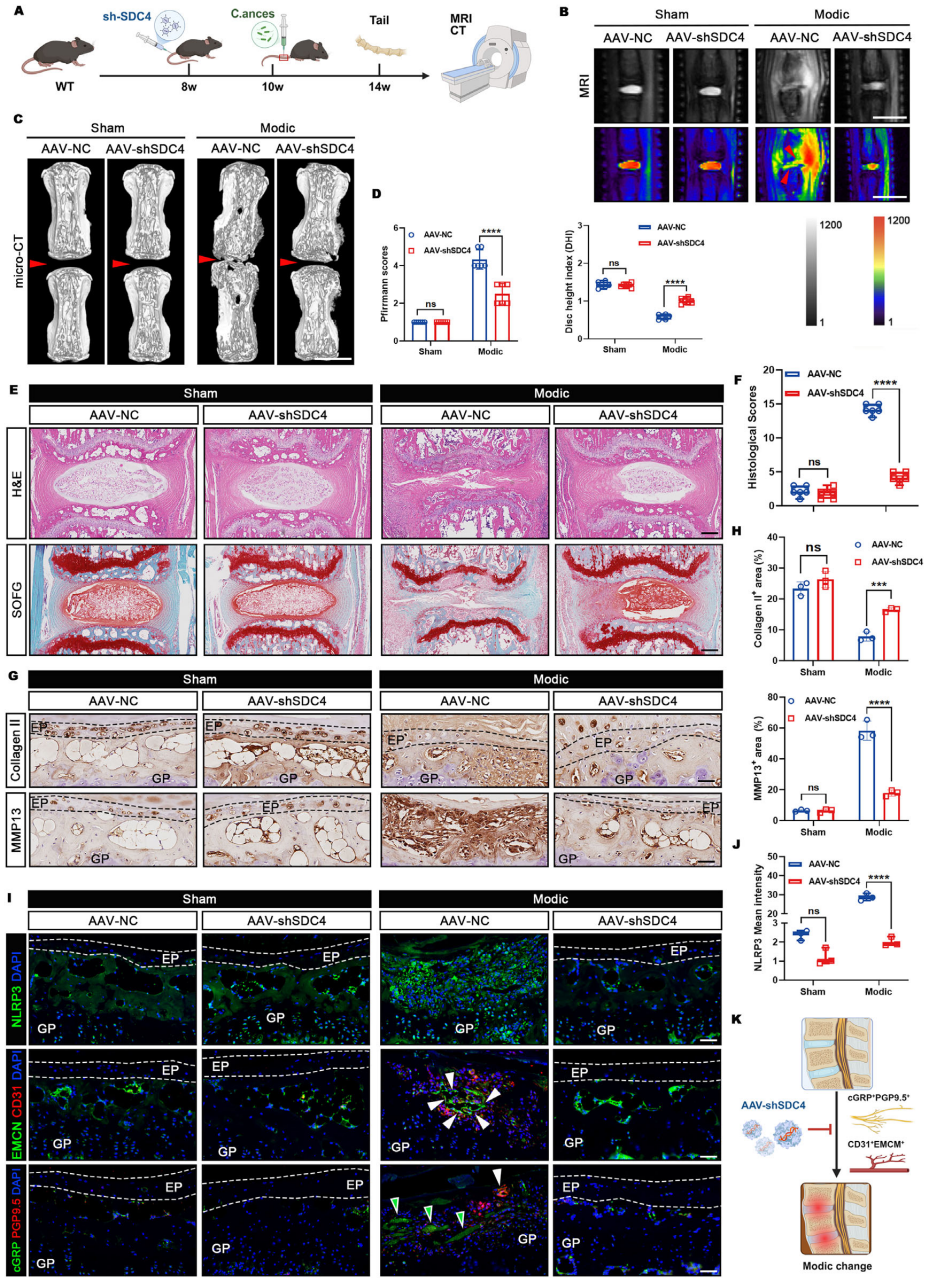
Targeting SDC4 alleviates MCs progression. (A) Schematic of the experimental timeline for locally injected endplate‐targeted AAV‐shSDC4 to mitigate C. acnes‐induced MCs in mice. 8‐week‐old male C57BL/6 mice received injections of AAV‐shSDC4 (with AAV‐NC as the control) into their caudal vertebrae. Two weeks later, an MC model was induced by C. acnes. All mice were sacrificed, and caudal vertebral samples were collected 4 weeks after modeling. (B) Representative images of MRI. The corresponding pseudocolor images were shown below. Typical features of Modic changes are marked with red arrows. Scale bar = 1 mm. (C, D) Representative micro‐CT images along with the disc height index and Pfirrmann grade (*n* = 6). The affected segments are marked by red arrows. Scale bar = 1 mm. (E, F) Representative images of H&E and SOFG staining, along with the histological scores (*n* = 6). Scale bar = 200 µm. (G, H) Representative immunohistochemical staining images for Collagen II and MMP13 and statistical analysis of the percentage of positive area (*n* = 3). Scale bar = 40 µm. (I) Representative images and statistical analysis of tissue immunofluorescence staining for NLRP3 inflammasome, vascular endothelial cells (CD31 and EMCN), and nerve fiber (cGRP and PGP9.5). White arrows indicate double‐positive cells. Scale bar = 40 µm. (J) statistical analysis of NLRP3 mean fluorescence intensity (*n* = 3). (K) Schematic diagram depicting the therapeutic role of SDC4 targeting in endplate chondrocytes for alleviating MCs. Statistical analyses were determined by two‐way ANOVA (D, F, H, J). ns indicated no statistical difference, ^*^ *p* < 0.05, ^**^ *p* < 0.01, ^***^ *p* < 0.001, and ^****^ *p* < 0.0001. Data were presented as mean ± SD.

To examine the detailed microstructural changes, we performed histological staining on tail vertebra sections. H&E and SOFG staining revealed that SDC4 knockdown ameliorated cartilaginous endplate destruction, reduced loss of proteoglycans in the nucleus pulposus and extracellular matrix, and resulted in lower histological scores (Figure 7E,F). Masson staining showed that SDC4 knockdown alleviated the disorganization of the annulus fibrosus induced by MCs (Figure S10C). Immunohistochemistry for Collagen II and MMP13 indicated that SDC4 knockdown preserved collagen II content, suppressed MMP13 upregulation, and rebalanced local anabolic and catabolic metabolism in the disc (Figure 7G,H; Figure S10D). Furthermore, the AAV‐shSDC4 group exhibited reduced levels of the NLRP3 inflammasome, as well as less vascular invasion and nerve fiber ingrowth (Figure 7I,J; Figure S10E). Thus, beyond ameliorating endplate degeneration, SDC4 knockdown effectively modulates the local disc microenvironment, reducing immune cell infiltration and potentially mitigating pathological pain (Figure 7K).

## 3 Discussion

Endplate inflammation is a degenerative spinal disorder involving vertebral endplate inflammation, typically manifesting as deep, dull low back pain corresponding to the affected spinal level. MRI serves as the gold standard for diagnosis, with Modic type I changes—seen as low signal intensity on T1‐weighted images and high signal on T2‐weighted and fat‐suppressed sequences—indicating bone marrow edema and an acute inflammatory state [43, 44]. As the condition progresses, it may transition to chronic stages dominated by fatty infiltration or sclerosis [45, 46, 47]. Clinical management is challenged by its multifactorial pathogenesis, the limited and transient efficacy of conservative therapies, and the invasive risks of surgical interventions [5, 48]. Thus, elucidating its underlying mechanisms is essential for developing targeted treatments.

Our group has been investigating the immune microenvironment and regulatory networks in MCs. In this study, we identified a marked expansion of THBS1‐positive macrophages within the MCs niche. These cells displayed pro‐inflammatory, pro‐proliferative, and pro‐angiogenic properties, implicating them in disease progression. Previous research in the skeletal system has shown that THBS1 knockout mice exhibit increased bone mass, thickened cortices, and reduced osteoclast differentiation and activity, underscoring the importance of THBS1 in bone homeostasis, including the maintenance of bone matrix integrity and regulation of osteoclastogenesis [49, 50]. Additionally, mechanical loading activates Piezo1, inducing periosteal CD68^+^ F4/80^–^ myeloid cells to differentiate into CD68^+^ F4/80^+^ macrophages, which in turn express and secrete THBS1, activating TGβ1 and recruiting osteoprogenitor cells—a process vital for fracture repair [51]. Here, C. acnes and its metabolites drive both THBS1 expression and M1‐like polarization in macrophages, and this specific subset promotes inflammation and tissue destruction. Crucially, macrophage‐specific THBS1 knockout attenuated C. acnes‐induced MCs in mice, highlighting its potential as a therapeutic target for MCs immunotherapy.

Beyond its known interactions with integrins, TGFβ1, CD36, and CD47, we found through single‐cell ligand‐receptor analysis that SDC4 is the critical receptor for THBS1 in the context of MCs. SDC4 expression is reportedly upregulated in chondrocytes from arthritis patients, and its knockout protects mice against arthritis‐related aggrecan loss [52, 53]. Furthermore, an antibody targeting the SDC4 dimerization domain significantly reduced IL1R1 expression in arthritic fibroblasts both in vitro and in rheumatoid arthritis models [54]. However, the specific role and mechanistic basis of SDC4 in MCs have remained elusive. Here, we demonstrate that THBS1 binding to SDC4 activates the downstream p38 MAPK pathway, leading to transcriptional upregulation of NLRP3 and subsequent inflammatory cytokine release, which ultimately drives degeneration of the cartilaginous endplate and microenvironmental deterioration. Importantly, targeted knockout of SDC4 in chondrocytes alleviated C. acnes‐induced MCs, indicating SDC4 as a critical downstream therapeutic target for mitigating endplate inflammation. To some extent, the role of THBS1 derived from endplate chondrocytes under physiological conditions or in Modic changes remains unclear, which is also a limitation of this study.

By profiling the MCs immune microenvironment, we established THBS1^+^ macrophages as a key degenerative subset and identified SDC4 as its critical receptor, highlighting both as promising therapeutic targets. These findings highlight THBS1 and SDC4 as promising targets for the immunotherapy of MCs. To fully realize this clinical potential, key mechanistic gaps must be addressed. These include elucidating potential cross‐talk between THBS1^+^ macrophages and other immune cells, and determining whether neutrophils, despite stable THBS1 expression, undergo functional changes that contribute to pathology. A deeper understanding of these aspects will critically inform the development of effective THBS1/SDC4‐directed treatments.

## 4 Conclusion

Our findings demonstrate that the progression of MCs is driven by THBS1^+^ macrophages, which promote endplate degeneration through the THBS1‐SDC4‐p38‐NLRP3 signaling axis. Consequently, therapeutic strategies targeting the THBS1/SDC4 interaction emerge as a promising immunotherapeutic approach.

## 5 Materials and Methods

*Sex as a biological variable*. Our study examined both male and female mice and humans.

### 5.1 Reagents and Antibodies

Hela cells were obtained from the National Collection of Authenticated Cell Cultures, Chinese Academy of Sciences. M‐CSF was obtained from R&D Systems (Minneapolis, Minnesota, USA). Antibodies of CD11b (14‐0112‐82) and THBS1 (MA5‐13390) were purchased from Invitrogen (California, USA). Antibodies of F4/80 (sc‐377009), CD31 (sc‐376764), cGRP (sc‐57053), P38 (sc‐7972), and p‐P38 (sc‐7973) were purchased from Santa Cruz Biotechnology (Texas, USA). Antibodies of Aggrecan (ET1704‐57), NLRP3 (ET1610‐93), and IL18 (HA721768) were purchased from HUABIO (Hangzhou, China). Antibodies of Adamts5 (ab41037) and MMP3 (ab52915) were purchased from Abcam. Antibodies of SOX9 (#82630), JNK (#9252), p‐JNK (#9255), Erk (#5013), p‐Erk (#4370), and Cleaved Caspase1 (#89332) were purchased from Cell Signaling Technology. Antibody of IL1β (A22257) was purchased from ABclonal. Antibodies of β‐actin (66009‐1‐Ig), EMCN (67854‐1‐Ig), PGP9.5 (14730‐1‐AP), and Caspase1 (22915‐1‐AP) were purchased from Proteintech (Wuhan, China). DAPI (C0065) was a product of Solarbio (Beijing, China). Antibody of Collagen II (bs‐0709R) was purchased from Bioss (Beijing, China). Antibody of Flag (M185‐3L) was purchased from MBL (Beijing, China). Goat anti‐Mouse HRP (FDM007) and Goat anti‐Rabbit HRP (FDR007) were purchased from Fudebio‐tech (Hangzhou, China). Anti‐Rabbit IgG Fab2 Alexa Fluor 488 Conjugate antibody and Anti‐Mouse IgG Fab2 Alexa Fluor 555 Conjugate antibody were purchased from Cell Signaling Technology (4412S and 4409S). Alpha‐modified Eagle's medium (α‐MEM), Dulbecco's modified Eagle's medium (DMEM), and fetal bovine serum (FBS) were purchased from Gibco (USA).

### 5.2 Mice

*Lysm‐Cre* mice were provided by Prof. Xiaojian Wang (Institute of Immunology, Zhejiang University School of Medicine, China). THBS1*^f/f^* mice were purchased from Cyagen Biosciences Inc. In all experiments, Lysm‐*Cre*; THBS1*^f/f^* mice were compared with their control littermates.

### 5.3 Single‐Cell Sequencing Analysis of MCs

Raw 10× Genomics data were processed using Seurat (v4). Cells expressing fewer than 300 genes, or genes detected in fewer than three cells, were excluded. Additional filtering was performed based on mitochondrial (≤20%), ribosomal (≤100%), and hemoglobin gene (≤20%) content. Data were log‐normalized, and the top 3000 variable genes were identified. Batch effects were corrected using anchor‐based integration. The integrated dataset was scaled and subjected to PCA, with the top 10 principal components used for clustering. Cluster resolution was optimized using clustree analysis, yielding seven major cell populations annotated based on canonical marker genes. Monocytes were further extracted and re‐clustered to identify classical, intermediate, non‐classical monocytes, and macrophage‐like subsets. UMAP and t‐SNE were used for visualization. Expression of THBS1 and SDC4 was visualized using FeaturePlot, and group comparisons were performed using Student's *t*‐test, one‐way ANOVA, or non‐parametric tests, as appropriate.

### 5.4 Cell Culture

Endplate Chondrocytes (EPs) Extraction: Rat EP tissues were isolated from lumbar discs and then cut into small pieces (<1 mm^3^ in size). After incubation with type II collagenase (0.2 mg/mL) (Sigma–Aldrich, USA) for 8 h at 37°C, the cells were harvested and centrifuged at 800 rpm for 5 min. The cells were resuspended with DMEM with 10% FBS.

Bone marrow‐derived macrophages (BMMs) extraction: femur and tibia bone marrow from 8‐week‐old C57BL/6J mice were extracted using a syringe, followed by culturing in α‐MEM supplemented with 10% FBS, 1% penicillin‐streptomycin, and 50 ng/mL M‐CSF. The cultures were maintained in a humidified atmosphere of 95% air and 5% CO_2_ at 37°C with medium renewal every two days until reaching pre‐osteoclast cells at 90% confluency.

### 5.5 High Density Chondrocytes Culture

A suspension of endplate chondrocytes was prepared at a high density following digestion and centrifugation procedures. A 20µL aliquot of the cell suspension was seeded into the center of each well in a 6‐well plate and cultured for 2 h under standard conditions (37°C, 5% CO_2_) to allow for cell attachment. Complete medium was then carefully added to each well to continue the culture. Macrophages were seeded into the upper chamber of a Transwell insert and co‐cultured with the chondrocytes for 48 h. After co‐culture, the cells were fixed with 4% paraformaldehyde for 15 min, washed with PBS, and stained with Safranin O solution for 30 min, followed by a PBS wash to remove non‐specific staining.

### 5.6 Human Tissue From Normal and Intervertebral Disc Degeneration Patients

Combined with clinical imaging data, endplate tissues were collected from patients undergoing anterior corpectomy and decompression for lumbar burst fractures as the patient control group. During lumbar interbody fusion surgery, partial endplate tissues from regions exhibiting different types of Modic changes were obtained. Informed consent was obtained from either the patient or from next of kin.

### 5.7 Western Blot

Total protein was extracted from adherent cells using RIPA lysate, followed by centrifugation at 12000 g for 15 min to collect the supernatant. The proteins were separated via 10% SDS‐PAGE and transferred onto PVDF membranes. Following a 1‐h blocking step with 5% skim milk powder at room temperature, the cells were subjected to overnight incubation with primary antibody at 4°C and subsequent incubation with secondary antibody for 1 h on a shaker. Protein strips were then exposed to ECL solution using a LAS‐4000 Science Imaging System (Fujifilm, Tokyo, Japan), and acquired images were subsequently analyzed and processed using Image J.

### 5.8 RNA Isolation and Quantitative RT‐PCR

CTotal RNA was extracted using the TRIzol reagent extraction protocol, followed by reverse transcription into cDNA using the HiFiScript cDNA Synthesis Kit (CW2569, CWBIO). The RT‐qPCR amplification reaction was performed utilizing Hieff qPCR SYBR Green Master Mix (11201ES08, Yeasen). The mRNA expression levels of the target genes were quantified by calculating Ct (threshold cycle) values and normalized to β‐actin levels. Primer sequences were listed in Tables 1 and 2.

**TABLE 1:** Mouse sequences of the primers used for RT‐PCR.

Mouse genes	Forward (5’‐3’)	Reverse (5’‐3’)
*Thbs1*	GGTAGCTGGAAATGTGGTGCGT	GCACCGATGTTCTCCGTTGTGA
*Il1β*	TGGACCTTCCAGGATGAGGACA	GTTCATCTCGGAGCCTGTAGTG
*Il6*	TACCACTTCACAAGTCGGAGGC	CTGCAAGTGCATCATCGTTGTTC
*Tnfα*	GGTGCCTATGTCTCAGCCTCTT	GCCATAGAACTGATGAGAGGGAG
*Cd80*	CCTCAAGTTTCCATGTCCAAGGC	GAGGAGAGTTGTAACGGCAAGG
*Cd86*	ACGTATTGGAAGGAGATTACAGCT	TCTGTCAGCGTTACTATCCCGC
*iNOS*	GAGACAGGGAAGTCTGAAGCAC	CCAGCAGTAGTTGCTCCTCTTC
*Cxcl1*	TCCAGAGCTTGAAGGTGTTGCC	AACCAAGGGAGCTTCAGGGTCA
*Cd206*	GTTCACCTGGAGTGATGGTTCTC	AGGACATGCCAGGGTCACCTTT
*Arg1*	CATTGGCTTGCGAGACGTAGAC	GCTGAAGGTCTCTTCCATCACC
*Cd163*	GGCTAGACGAAGTCATCTGCAC	CTTCGTTGGTCAGCCTCAGAGA
*Il10*	CGGGAAGACAATAACTGCACCC	CGGTTAGCAGTATGTTGTCCAGC
*Tgfβ1*	TGATACGCCTGAGTGGCTGTCT	CACAAGAGCAGTGAGCGCTGAA
*β‐Actin*	ACAGCAGTTGGTTGGAGCAA	ACGCGACCATCCTCCTCTTA

**TABLE 2:** Rat sequences of the primers used for RT‐PCR.

Rat genes	Forward (5’‐3’)	Reverse (5’‐3’)
*Aggrecan*	GCAGCACAGACACTTCAGGA	CCCACTTTCTACAGGCAAGC
*CollagenII*	CTGGAAAAGCTGGTGAAAGG	GGCCTGGATAACCTCTGTGA
*Sox9*	GGATGTCAAAGCAACAGGCG	ATGTGCGTTCTCTGGGACTG
*Adamts5*	AGTACAGTTTGCCTACCGCC	GATTTGCCGTTAGGTGGGCA
*Mmp3*	CAAGTCCTCCACAGACCTGG	GCTGACTGCATCGAAGGACA
*Mmp13*	CCTGGAGCCCTGATGTTTC	TGGGTCACACTTCTCTGGTG
*Sdc4*	TGTTGCTCCTCGGAGGTTTC	GGAACCCGACAGCTCAAAGT
*Nlrp3*	TCTGCATGCCGTATCTGGTT	AGGGTACCCCATAGACTGGC
*Il1β*	AGCTTCAGGAAGGCAGTGTC	TCAGACAGCACGAGGCATTT
*Il18*	GGCACAGCCTCTCAGTTGGA	CTCATCGTTGTGGGGACAGC
*β‐Actin*	CTATGAGGGTTACGCGCTCC	ATGTCACGCACGATTTCCCT

### 5.9 Safranin O‐Fast Green (SOFG)

The mouse tails were dissected and fixed in 4% paraformaldehyde for 48 h, followed by decalcification using EDTA for 14 days. Subsequently, the samples underwent dehydration, paraffin embedding, and sectioning into 5 µm thick slices. The sections were deparaffinized with xylene and rehydrated through a graded ethanol series. SOFG staining was then performed according to standard protocols.

### 5.10 Masson Staining

After deparaffinization, sections are stained with Weigert's iron hematoxylin for 8 min, differentiated with acidic ethanol for 15 s, and rinsed. They are then treated with Masson staining solution for 5 min, followed by washing. Next, staining is performed with ponceau fuchsin for 5 min, rinsed with weak acid working solution, treated with phosphomolybdic acid, rinsed again with weak acid solution, and stained with aniline blue for 2 min. After a final weak acid rinse, sections are dehydrated and mounted with neutral gum.

### 5.11 Immunofluorescence Staining

The medium was aspirated, and the cells were fixed with 4% paraformaldehyde (G1101, Servicebio) for 15 min after being washed three times with PBS. 0.1% Triton X‐100 for 15 min at room temperature, and QuickBlock immunostaining blocking solution (P0260, Beyotime) was then applied at room temperature for 1 h. The blocking solution was discarded, and the corresponding primary antibody was added for overnight incubation at 4°C. The plates were washed thrice with PBS, each time for 10 min, followed by incubation with fluorescent secondary antibody and DAPI in a dark room at room temperature for an hour. Finally, the slides were sealed using an anti‐quenching agent. Fluorescence images were captured using a Nikon A1 Ti confocal microscope and analyzed through Image J.

Following dewaxing of the tissue sections, antigen retrieval was performed by overnight soaking in sodium citrate buffer at 55°C. After slowly cooling to room temperature, QuickBlock immunostaining sealer was applied and allowed to cure for 1 h at room temperature before addition of primary antibody and overnight incubation at 4°C. The slides were subjected to three washes with TBST prior to incubation with fluorescent secondary antibody and DAPI for 1 h at room temperature, followed by another three washes with TBST. The anti‐quenching agent was hermetically sealed before fluorescence scanning.

### 5.12 Immunohistochemistry Staining

For immunohistochemistry staining, sections were submerged with sodium citrate at 55°C overnight for antigen retrieving. After being blocked with BSA for 1 h, the sections were incubated with anti‐Collagen II antibody at 4°C overnight and washed with PBS for three times and incubated with anti‐mouse secondary antibody (ZsBio, China). Sections were washed with PBS for three times and stained with DAB solution (ServiceBio, China) at RT for 5–10 min. The images were captured by a microscope (Nikon, Japan).

### 5.13 Transfection of SDC4 siRNA

Lipofectamine RNAiMAX (13778150, Invitrogen) was utilized for transfection in accordance with the manufacturer's instructions. siRNAs targeting SDC4 were synthesized by Tsingke, China. SDC4 siRNA: 5’‐3’ GGCAGAUACUUCUCUGGAGdTdT; 3’‐5’ dTdTCCGUCUAUGAAGAGACCUC.

### 5.14 RNA‐seq and Bioinformatic Analysis

Total RNA from 1 × 10^6^ EPs per well was extracted using the TRIzol reagent following the manufacturer's instructions. Paired‐end sequencing was performed on an Illumina HiSeq 4000 (LC Bio, China) according to the vendor's recommended protocol after library preparation. The number of reads mapped to each gene is presented as fragments per kilobase of transcript per million fragments mapped (FPKM) values. Differentially expressed genes were selected based on a log2 (fold change) >1 or log2 (fold change) <−1 and statistical significance (*P* < 0.05). Bioinformatic analysis was conducted to identify significant pathways and gene sets between the NC and SDC4 siRNA group based on normalized gene expression data.

### 5.15 Micro‐CT Scanning and Analysis

Mice were euthanized to obtain spine, femur, and tibia tissues. The bone tissues were subsequently fixed in 4% paraformaldehyde for 48 h before micro‐CT scanning (SkyScan1275, Bruker, USA) with the following parameters: a source voltage of 50 kV, a source current of 450 µA, an aluminum (AI) filter thickness of 0.5 mm, a pixel size of 9 µm, and a rotation step of 0.4°. Furthermore, the acquired images were reconstructed using NRecon software (Bruker micro‐CT, Kontich, Belgium). Total porosity, Endplate volume, Porosity volume, and Disc height parameters were evaluated with CTAn program (Bruker micro‐CT, Kontich, Belgium).

### 5.16 Modic Changes (MCs) Model

Our team has previously established and validated an animal model of Modic changes by local injection of C. acnes into the endplate [17, 28, 29, 30]. Under fluoroscopic guidance, a percutaneous caudal endplate puncture technique was used to establish a C. acnes infection‐induced Modic change model in mouse caudal vertebrae. The 6th/7th caudal intervertebral disc level was selected as the model disc sample. The control group received an equal volume of sterile saline. After anesthesia, the mice were shaved and disinfected. Under anteroposterior (AP) and lateral fluoroscopic guidance, a 29‐gauge needle was inserted at the lower one‐third of the vertebral body, advancing with a rotational motion at a horizontal angle of approximately 60° toward the vertebral body. When the needle tip reached the midline of the vertebral body below the endplate on AP view, and the anterior 2/5 to 3/5 junction of the vertebral body on lateral view, 10µL of C. acnes (ATCC#6919, 1.6 × 10^7^ CFU/mL) was injected into the endplate region of the 6/7 caudal disc. The needle was then safely withdrawn. At 4 weeks post‐injection, model establishment was evaluated by imaging (e.g., MRI and micro‐CT).

### 5.17 Flow Cytometry

Bone marrow cells from wild‐type (WT) and THBS1 knockout (THBS1 KO) mice were isolated, treated with red blood cell lysis buffer to remove erythrocytes, and washed three times with PBS. The cells were resuspended in PBS staining buffer containing 2% FBS, stained with F4/80‐FITC (BM8, Biolegend) antibody, and incubated for 20 min at 4°C in the dark, followed by three washes with PBS. For CD206 staining, CD206‐PE (C068C2, Biolegend) antibody was added directly, and the cells were incubated for 30 min at 4°C in the dark, washed, and resuspended for flow cytometry. For iNOS staining, after surface staining with F4/80, the cells were treated with fixation/permeabilization solution, then stained with iNOS‐PE (W16030C, Biolegend) antibody for 30 min at 4°C in the dark, washed, and resuspended for analysis. Appropriate isotype controls and single‐staining compensation controls were included in all steps.

### 5.18 ELISA Assay

Blood samples collected from wild‐type (WT) and THBS1 knockout (THBS1 KO) mice were allowed to stand at room temperature for 1 h, then centrifuged at 3000 rpm for 15 min to obtain serum. Serum THBS1 levels were measured using a THBS1 ELISA detection kit (SEA611Mu, Cloud‐Clone, Wuhan, China) according to the manufacturer's instructions.

### 5.19 Statistical Analysis

At least three independent sets of experiments were conducted in triplicate, and the results were presented as mean ± SD. Statistical analyses were performed using Graph Pad Prism 9.0 for Windows (Graph Pad Software, San Diego, CA, USA). In the statistical analysis, one‐way ANOVA and two‐way ANOVA were employed for multiple comparisons tests. Unpaired two‐tailed Student's *t*‐tests were utilized to compare means between two groups. Significance was determined at *p* < 0.05. ^*^ *p* < 0.05, ^**^ *p* < 0.01, ^***^ *p* < 0.001, and ^****^ *p* < 0.0001.

## Author Contributions

**Xiangxi Kong**, **Qize Xue**, **Jie Li**, and **Xiaoan Wei** performed the experiments, analyzed the data and edited the manuscript. Xiangxi Kong, Qize Xue, **Zimin Cai**, **Tao yang**, **Weishao Chen**, **Bohan Cai**, Xiaoan Wei, **Chengjun Yao** and **Jiayan Jin** performed mice experiments and revised the manuscript. **Junhui Liu**, **Bao Huang**, **Xuyang Zhang** conceived the project and revised the manuscript. **Fengdong Zhao**, **Zhi Shan**, **Haihao Wu** and **Jian Chen** conceived the project, designed the experiments and edited the manuscript. All authors discussed the results and contributed to the preparation of the manuscript.

## Funding

This work was supported by the National Natural Science Foundation of China [Grant Nos. 82572791, 82272521, 82302732] and Zhejiang Provincial Natural Science Foundation of China [LZ23H060002].

## Conflicts of Interest

The authors declare no conflicts of interest.

## Ethical Statements

All animal studies were approved by the Ethics Committee of Zhejiang University School of Medicine (ZJU20260020). All human studies were approved by the Ethical Review Board of Sir Run Run Shaw Hospital, Zhejiang University School of Medicine (2026‐0363).

## Supporting information




**Supporting File**: advs76318‐sup‐0001‐SuppMat.docx.

## Data Availability

The data that support the findings of this study are openly available in the Sequence Read Archive (SRA) at https://submit.ncbi.nlm.nih.gov/, reference number PRJNA1471691and PRJNA1471690.
