# Supplementary material for: THBS1+ Macrophages Exacerbate Modic Changes via SDC4‐Dependent Activation of NLRP3 Inflammasome
Source: Adv Sci (Weinh). 2026 Jun 26:e76318. Online ahead of print. doi: 10.1002/advs.76318 (PMC13336481; doi:10.1002/advs.76318)
Supplement: Supplementary file 1 — Supporting File: advs76318‐sup‐0001‐SuppMat.docx. [file ADVS-9999-e76318-s001.docx]

**THBS1^+^ Macrophages Exacerbate Modic Changes via SDC4-dependent Activation of NLRP3 Inflammasome**

*Xiangxi Kong^1,2^, Qize Xue^3^, Jie Li^4^, Haihao Wu^5^, Bao Huang^1,2^, Weishao Chen^6^, Zimin Cai^3^, Tao Yang^1,2^, Chengjun Yao^1,2^, Jiayan Jin^1,2^, Bohan Cai^1,2^, Xiaoan Wei^1,2^, Xuyang Zhang^1,2^, Junhui Liu^1,2^, Jian Chen^1,2,3*^, Zhi Shan^1,2*^, and Fengdong Zhao^1,2,3*^*


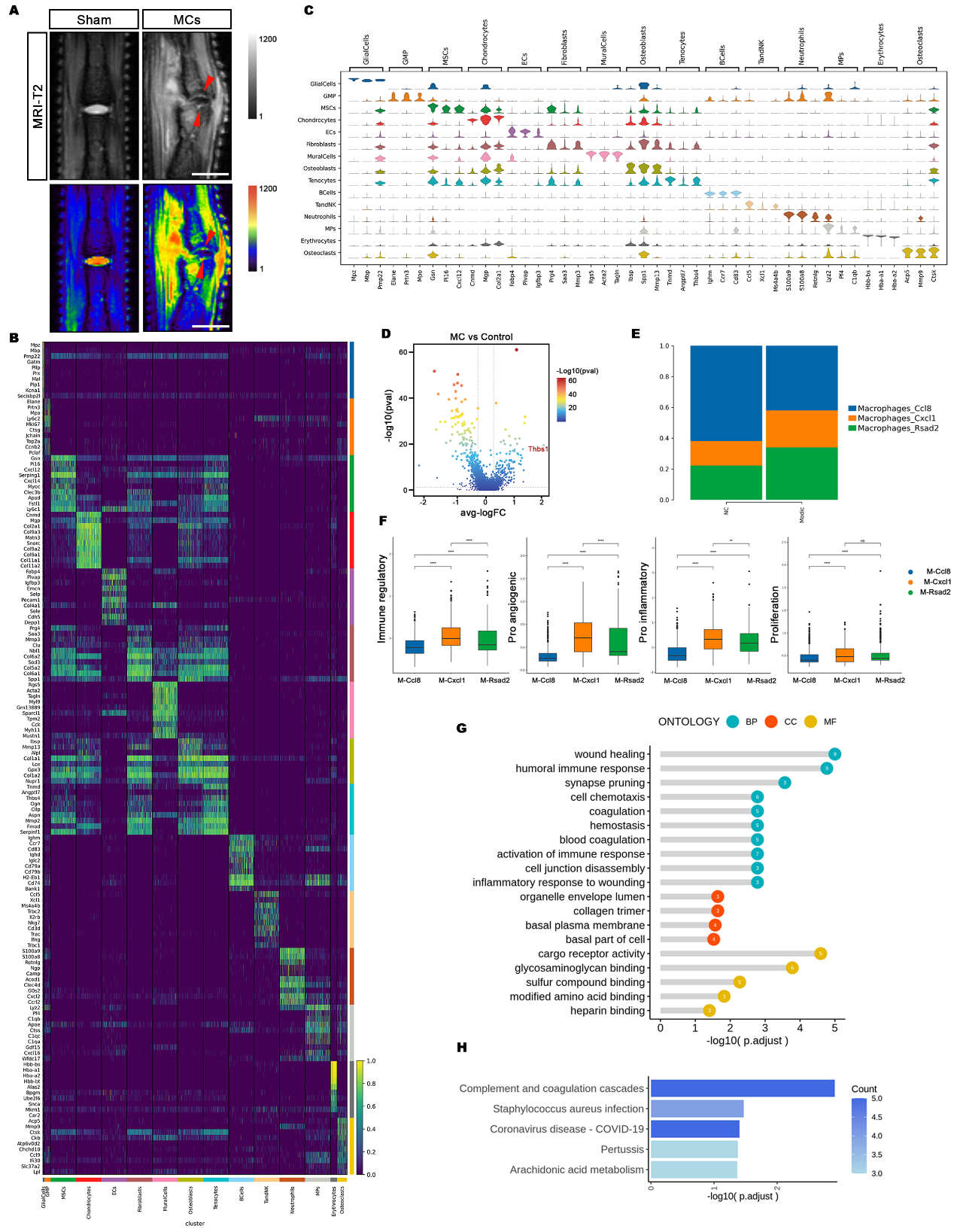


**Figure.S1. Single-cell RNA sequencing of MCs tissues identifies THBS1^+^ macrophages.** (A) Representative images of magnetic resonance imaging (MRI) of C. acnes-induced modic changes. The corresponding pseudocolor images were shown below. Typical features of Modic changes are marked with red arrows. Scale bar = 1mm. (**B**) Heatmap of genes of different cell subsets in MCs. (**C**) Violin plots of marker genes for distinct cell clusters. (**D**) Volcano plot of differentially expressed genes in MPs between the control and MCs groups. (**E**) Proportional abundances of distinct macrophage subsets in the control and MCs groups. (**F**) Functional characteristics (immune regulatory, pro-angiogenic, pro-inflammatory, and proliferation) of distinct macrophage subsets. (**G, H**) GO and KEGG enrichment analyses of down-regulated differentially expressed genes.


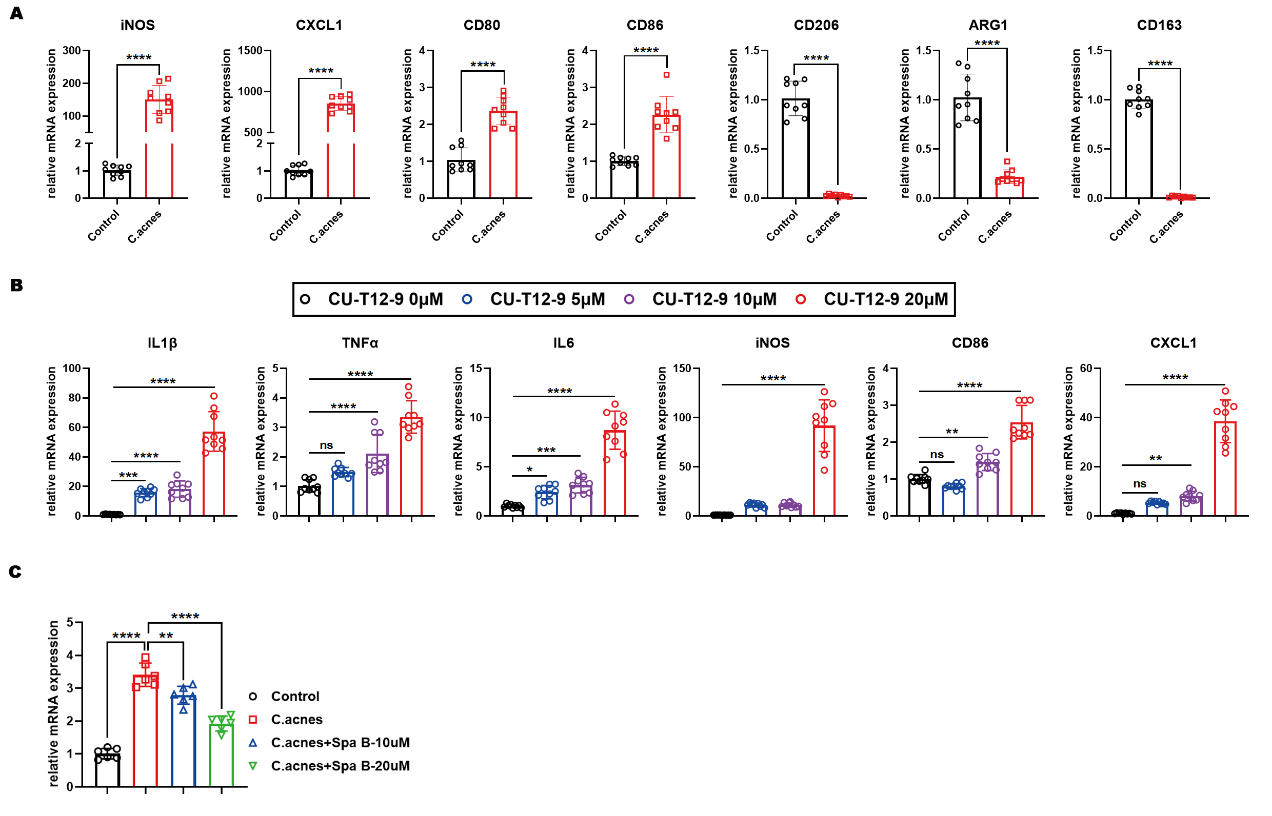


**Figure.S2. C. acnes and Its Metabolites Promote THBS1 Expression in Macrophages.** (**A**) RT-qPCR of macrophage polarization markers in BMMs stimulated with C. acnes for 24 hours (n=9). (**B**) RT-qPCR of TLRs target genes and M1 polarization markers in BMMs treated with different concentrations of TLR2 agonist CU-T12-9 for 24 hours (n=9). (**C**) RT-qPCR of THBS1 gene levels in BMMs treated with different concentrations of TLRs inhibitor Sparstolonin B for 24 hours (n=6). Statistical analyses were determined by two-tailed Student’s *t*-test (**A**) and one-way ANOVA (**B, C**). ns indicated no statistical difference, *P<0.05, **P<0.01, ***P<0.001, and ****P<0.0001. Data were presented as mean ± SD.


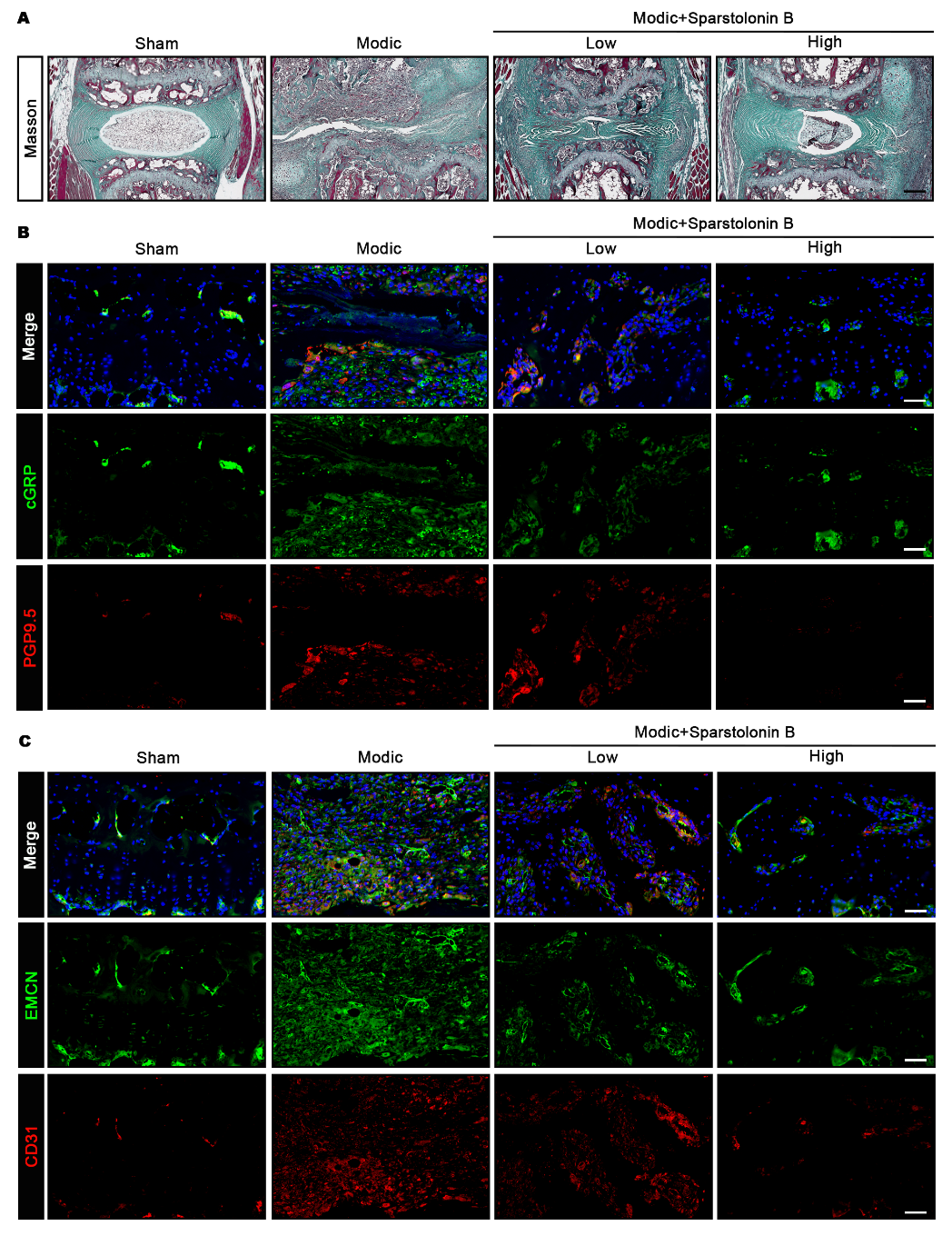


**Figure.S3. TLR2/4 Antagonism Suppresses Macrophage THBS1 Expression and Alleviates MCs.** (**A**) Representative images of Masson staining. Scale bar = 200μm. (**B**) Representative images of tissue immunofluorescence staining for nerve fiber (cGRP and PGP9.5). Scale bar = 40μm. (**C**) Representative images of tissue immunofluorescence staining for vascular endothelial cells (CD31 and EMCN). Scale bar = 40μm.


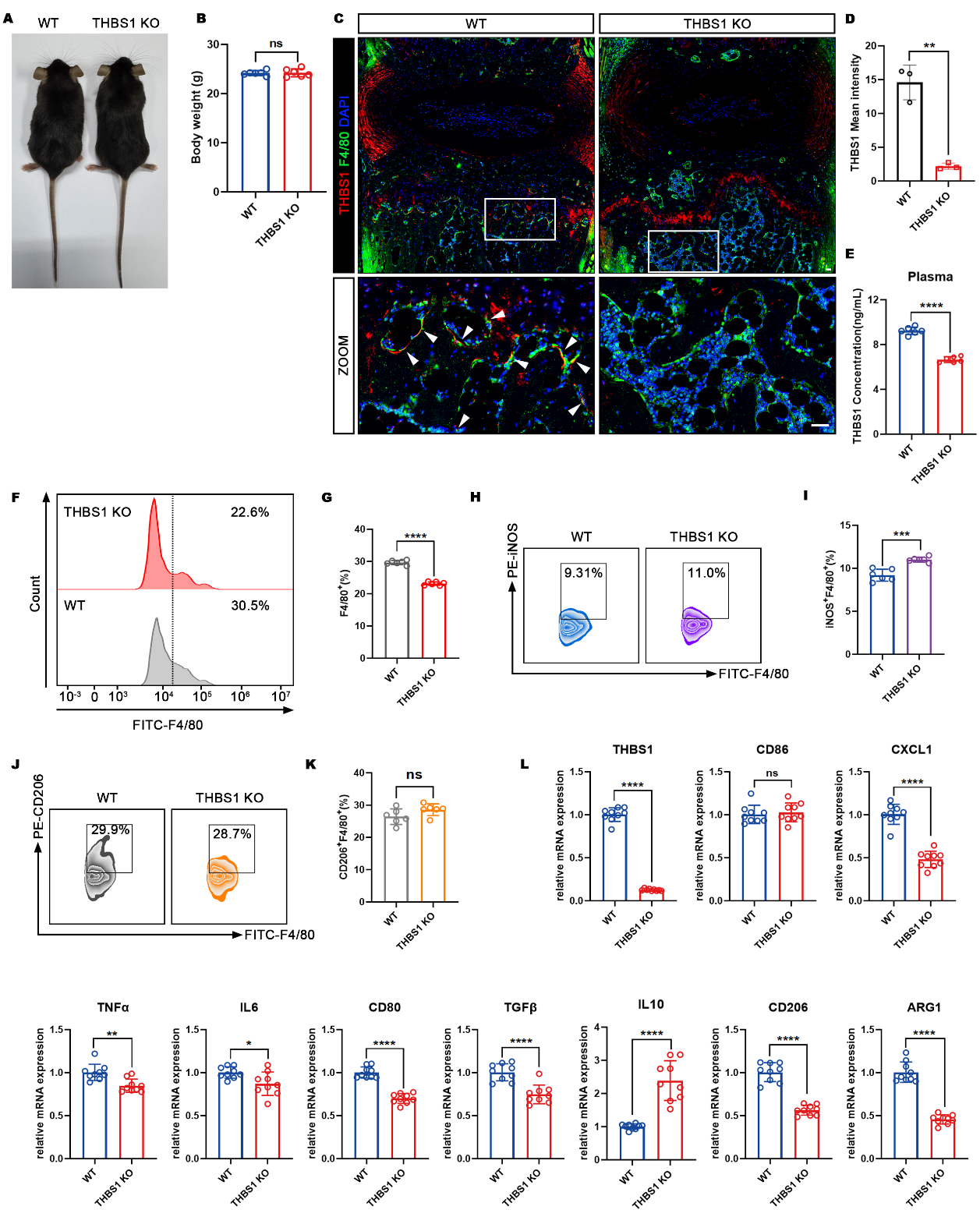


**Figure.S4. Macrophage-specific THBS1 knockout.** (**A**) Phenotype images of knockout mice. (**B**) Statistical analysis of the body weight of knockout mice (n=6). (**C-D**) Representative images of tissue immunofluorescence staining of THBS1 and F4/80. Statistical analysis of fluorescence intensity (n=3). White arrows indicate THBS1^+^F4/80^+^ double-positive cells. Scale bar = 40μm. (**E**) ELISA assay for THBS1 concentration levels in the serum of WT and THBS1 knockout mice (n=6). (**F-G**) Flow cytometric analysis of F4/80⁺ macrophages in the bone marrow (n=6). (**H-I**) Flow cytometric analysis of iNOS⁺ F4/80⁺ macrophages in the bone marrow (n=6). (**J-K**) Flow cytometric analysis of CD206⁺ F4/80⁺ macrophages in the bone marrow (n=6). (**L**). RT‑qPCR analysis of macrophage polarization‑related markers following THBS1 knockout (n=9). Statistical analyses were determined by two-tailed Student’s *t*-test (**B, D, E, G, I, K, L**). ns indicated no statistical difference, *P<0.05, **P<0.01, ***P<0.001, and ****P<0.0001. Data were presented as mean ± SD.


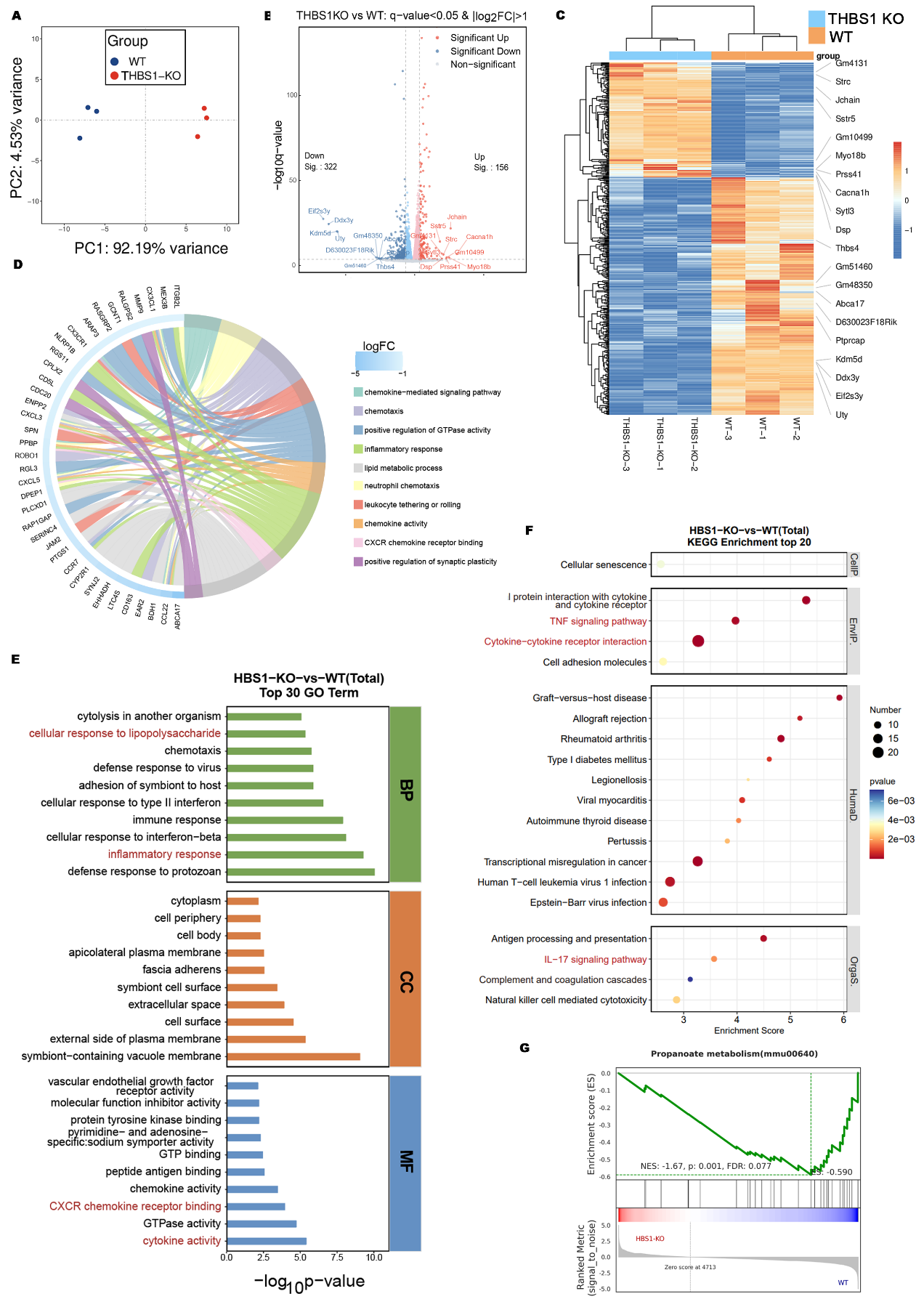


**Figure.S5. Transcriptomic analysis of THBS1-knockout BMMs upon C. acnes stimulation.** (**A**) Principal component analysis (PCA) of RNA-seq data from wild-type (WT) and Thbs1-knockout (KO) BMMs stimulated with C. acnes. (**B**) Volcano plot showing differentially expressed genes (DEGs) between THBS1 KO and WT BMMs. (**C**) Heatmap of DEGs illustrating distinct gene expression profiles between THBS1 KO and WT BMMs. (**D**) Chord plot of downregulated DEGs. (**E**) GO enrichment analysis of DEGs. (**F**) KEGG pathway enrichment analysis of DEGs. (**G**) GSEA of metabolism of propanoate.


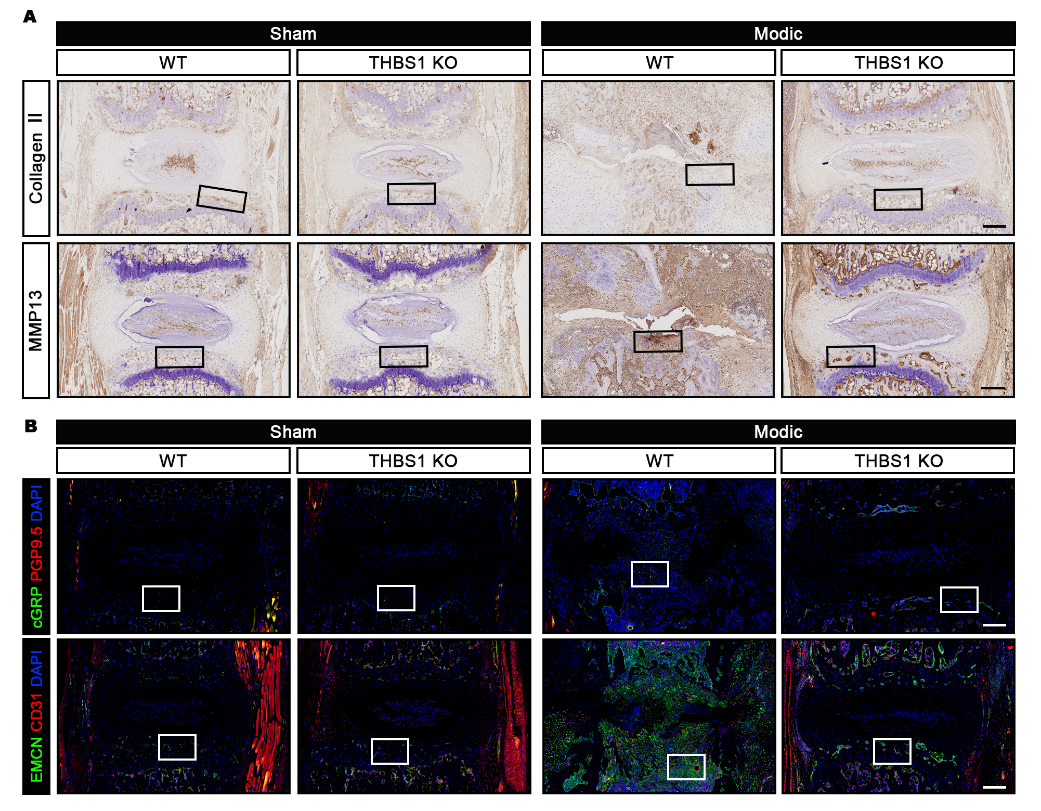


**Figure.S6. Macrophage-specific THBS1 knockout alleviates C. acnes-induced MCs.** (**A**) Representative low-magnification images of Collagen II and MMP13 immunohistochemical staining. Scale bar = 200μm. (**B**) Representative low-magnification images of tissue immunofluorescence staining for vascular endothelial cells (CD31 and EMCN) and nerve fiber (cGRP and PGP9.5). Scale bar = 200μm.


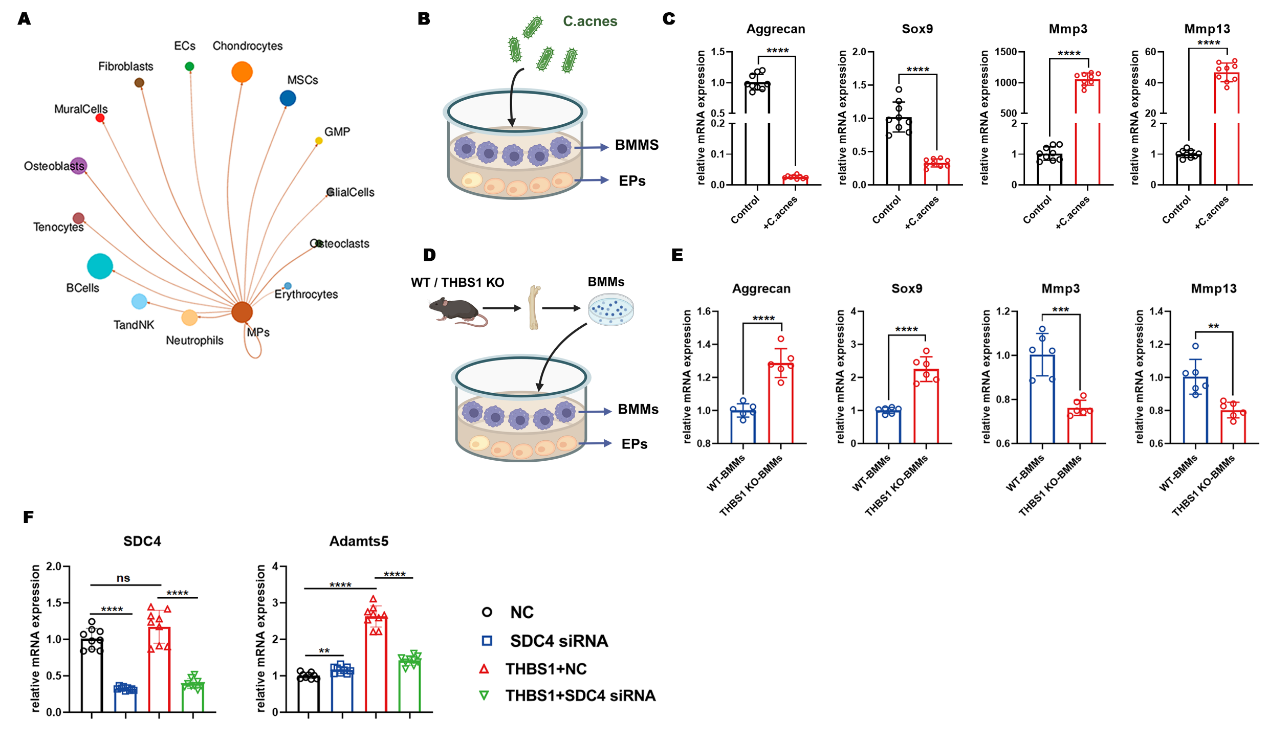


**Figure.S7. Identification of SDC4 on Chondrocytes as the Key Receptor of THBS1.** (**A**) CellChat analysis of interaction networks between macrophages and other cell types. (**B**) Schematic diagram of the co-culture system with C. acnes-stimulated macrophages and endplate chondrocytes. (**C**) RT-qPCR of anabolic and catabolic gene expression in endplate chondrocytes co-cultured with C. acnes-stimulated macrophages (n=9). (**D**) Schematic diagram of the co-culture system with THBS KO macrophages and endplate chondrocytes. (**E**) RT-qPCR of anabolic and catabolic gene expression in endplate chondrocytes co-cultured with THBS1-KO macrophages (n=6). (**F**) RT-qPCR of SDC4 and Adamts5 in chondrocytes transfected with SDC4 siRNA and treated with recombinant THBS1 protein (n=9). Statistical analyses were determined by two-tailed Student’s *t*-test (**C, E**) and one-way ANOVA (**F**). ns indicated no statistical difference, *P<0.05, **P<0.01, ***P<0.001, and ****P<0.0001. Data were presented as mean ± SD.


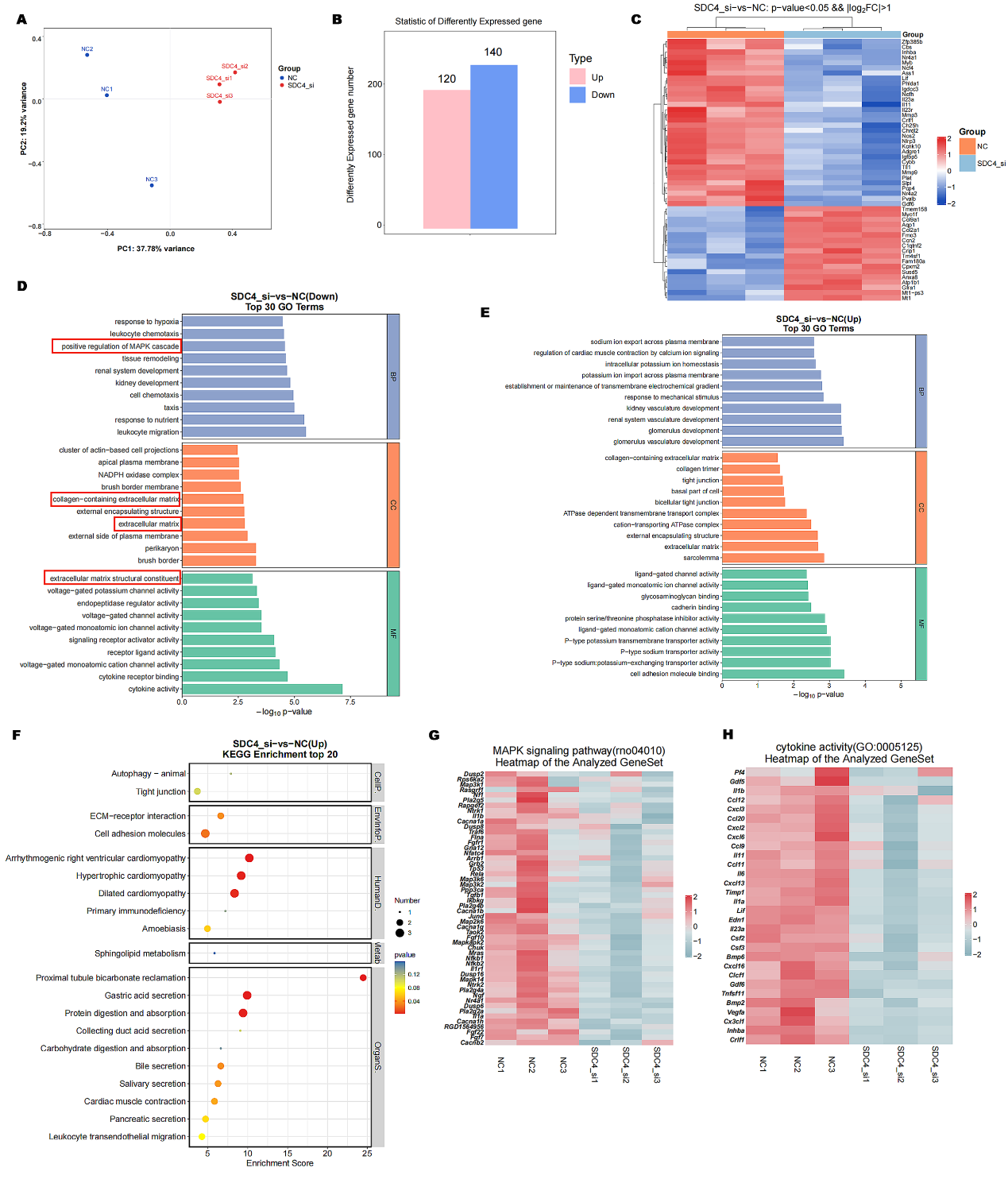


**Figure.S8. RNA sequencing of SDC4 siRNA-transfected endplate chondrocytes treated with recombinant THBS1 protein.** (**A**) Principal component analysis. (**B**) Bar graph showing the numbers of significantly differentially expressed genes. (**C**) Heatmap of the top 50 differentially expressed genes. (**D**) GO enrichment analysis of up-regulated differentially expressed genes. (**E**) GO enrichment analysis of down-regulated differentially expressed genes. (**F**) KEGG enrichment analysis of up-regulated differentially expressed genes. (**G, H**) Heatmap of differentially expressed genes related to the MAPK signaling pathway and cytokine activity.


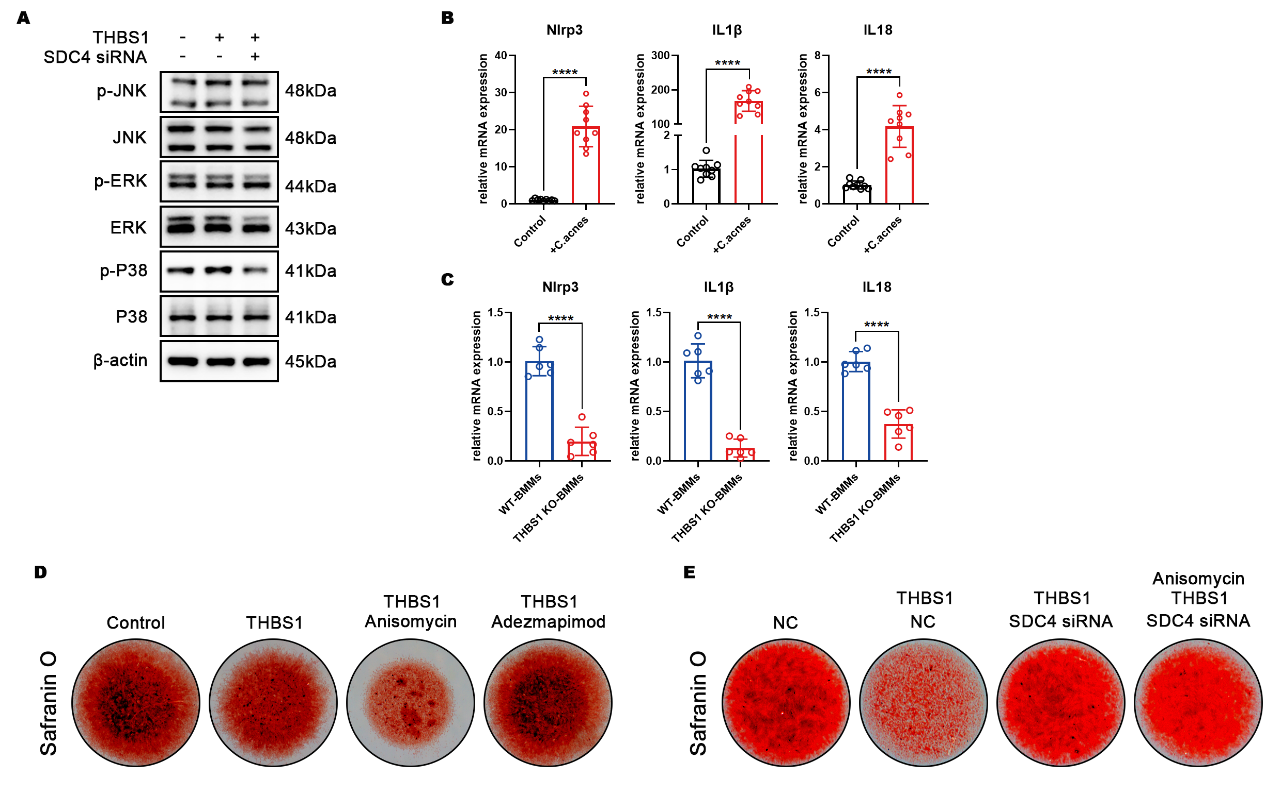


**Figure.S9. The SDC4-p38-NLRP3 Inflammasome Axis Promotes Degeneration of Endplate Chondrocytes.** (**A**) Western blot of the MAPK signaling pathway in SDC4 siRNA-transfected endplate chondrocytes treated with recombinant THBS1 protein. (**B**) RT-qPCR of inflammatory cytokine levels in endplate chondrocytes co-cultured with C. acnes-stimulated macrophages (n=9). (**C**) RT-qPCR of inflammatory cytokine levels in endplate chondrocytes co-cultured with THBS1 KO macrophages (n=6). (**D**) Safranin O staining of endplate chondrocytes in high-density culture treated with recombinant THBS1 protein and supplemented with a p38 agonist (anisomycin, 100nM) or a p38 inhibitor (adezmapimod, 20nM). (**E**) Safranin O staining of endplate chondrocytes in SDC4 siRNA‑transfected endplate chondrocytes treated with recombinant THBS1 protein and the p38 agonist anisomycin. Statistical analyses were determined by two-tailed Student’s *t*-test (**B, C**). ns indicated no statistical difference, *P<0.05, **P<0.01, ***P<0.001, and ****P<0.0001. Data were presented as mean ± SD.


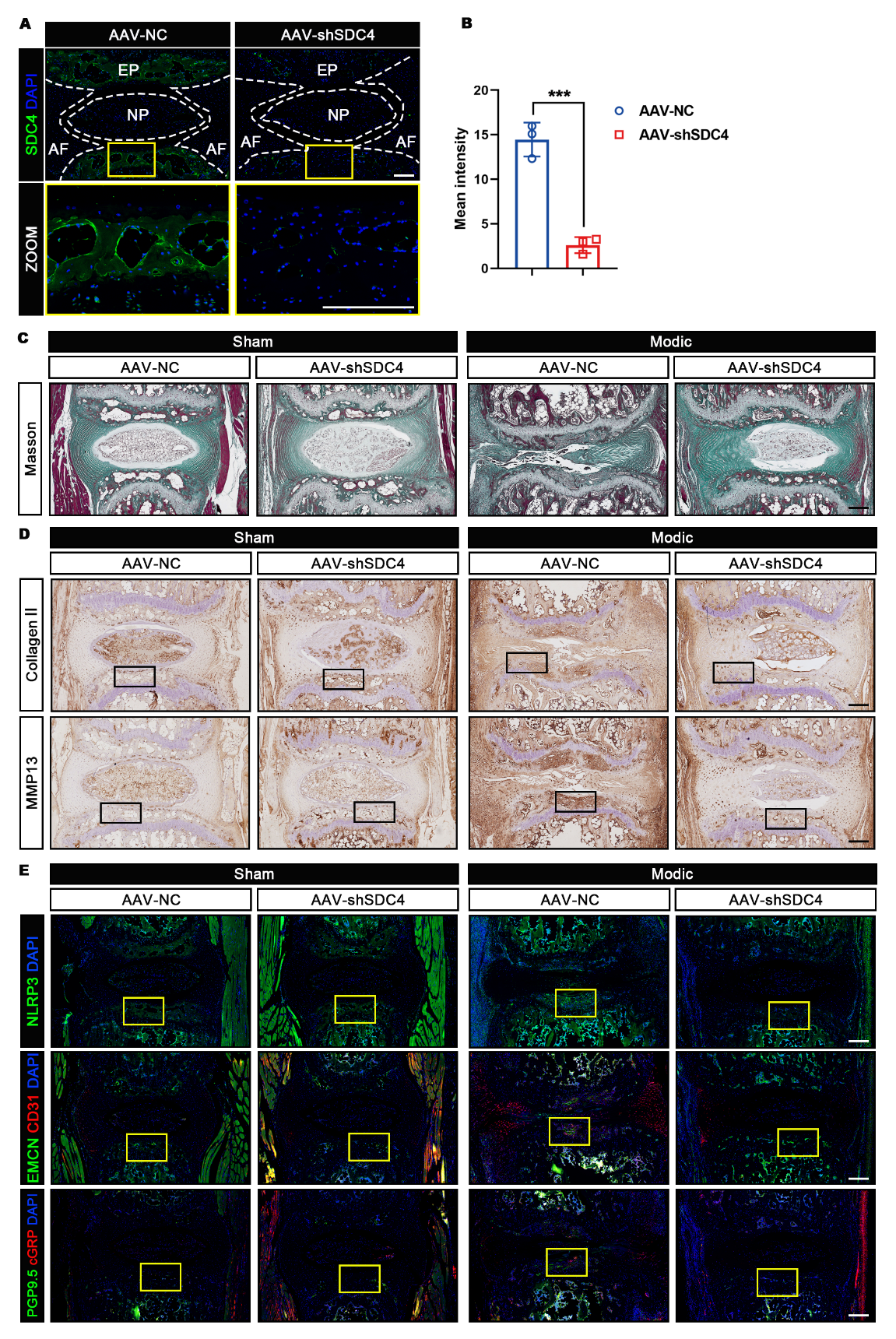


**Figure.S10. Targeting SDC4 alleviates MCs progression.** (**A, B**) Representative immunofluorescence images of SDC4 staining and corresponding quantitative analysis of fluorescence intensity (n=3). Scale bar = 200μm. (**C**) Representative image of Masson staining. Scale bar = 200μm. (**D**) Representative low-magnification images of Collagen II and MMP13 immunohistochemical staining. Scale bar = 200μm. (**E**) Representative low-magnification images of tissue immunofluorescence staining for NLRP3, vascular endothelial cells (CD31 and EMCN) and nerve fiber (cGRP and PGP9.5). Scale bar = 200μm. Statistical analyses were determined by two-tailed Student’s *t*-test (**B**). ns indicated no statistical difference, *P<0.05, **P<0.01, ***P<0.001, and ****P<0.0001. Data were presented as mean ± SD.
